# Novel Thiosemicarbazones Sensitize Pediatric Solid Tumor Cell-Types to Conventional Chemotherapeutics through Multiple Molecular Mechanisms

**DOI:** 10.3390/cancers12123781

**Published:** 2020-12-15

**Authors:** Silvia Paukovcekova, Jan Skoda, Jakub Neradil, Erika Mikulenkova, Petr Chlapek, Jaroslav Sterba, Des R. Richardson, Renata Veselska

**Affiliations:** 1 Department of Experimental Biology, Faculty of Science, Masaryk University, 61137 Brno, Czech Republic; silvia.paukovcekova@mail.muni.cz (S.P.); jan.skoda@sci.muni.cz (J.S.); jneradil@sci.muni.cz (J.N.); mikulenkova@mail.muni.cz (E.M.); chlapek@sci.muni.cz (P.C.); 2 International Clinical Research Center, St. Anne’s University Hospital, 65691 Brno, Czech Republic; 3 Department of Pediatric Oncology, University Hospital Brno and Faculty of Medicine, Masaryk University, 66263 Brno, Czech Republic; Sterba.Jaroslav@fnbrno.cz; 4 Griffith Institute for Drug Discovery, Centre for Cancer Cell Biology and Drug Discovery, Griffith University, Brisbane, Queensland 4111, Australia; d.richardson@griffith.edu.au

**Keywords:** thiosemicarbazones, DpC, Dp44mT, celecoxib, temozolomide, etoposide, combined anti-cancer treatment, osteosarcoma, medulloblastoma, neuroblastoma

## Abstract

**Simple Summary:**

Combination of chemotherapeutics for the treatment of childhood cancer can lead to the use of lower cytotoxic drug doses and better therapeutic tolerability (i.e., lower side effects) for patients. We discovered novel molecular targets of two lead thiosemicarbazone agents of the di-2-pyridylketone thiosemicarbazone class. These molecular targets include: cyclooxygenase, the DNA repair protein, O6-methylguanine DNA methyltransferase, mismatch repair proteins, and topoisomerase 2α. This research also identifies promising synergistic interactions of these thiosemicarbazones particularly with the standard chemotherapeutic, celecoxib.

**Abstract:**

Combining low-dose chemotherapies is a strategy for designing less toxic and more potent childhood cancer treatments. We examined the effects of combining the novel thiosemicarbazones, di-2-pyridylketone 4-cyclohexyl-4-methyl-3-thiosemicarbazone (DpC), or its analog, di-2-pyridylketone-4,4-dimethyl-3-thiosemicarbazone (Dp44mT), with the standard chemotherapies, celecoxib (CX), etoposide (ETO), or temozolomide (TMZ). These combinations were analyzed for synergism to inhibit proliferation of three pediatric tumor cell-types, namely osteosarcoma (Saos-2), medulloblastoma (Daoy) and neuroblastoma (SH-SY5Y). In terms of mechanistic dissection, this study discovered novel thiosemicarbazone targets not previously identified and which are important for considering possible drug combinations. In this case, DpC and Dp44mT caused: (1) up-regulation of a major protein target of CX, namely cyclooxygenase-2 (COX-2); (2) down-regulation of the DNA repair protein, O^6^-methylguanine DNA methyltransferase (MGMT), which is known to affect TMZ resistance; (3) down-regulation of mismatch repair (MMR) proteins, MSH2 and MSH6, in Daoy and SH-SY5Y cells; and (4) down-regulation in all three cell-types of the MMR repair protein, MLH1, and also topoisomerase 2α (Topo2α), the latter of which is an ETO target. While thiosemicarbazones up-regulate the metastasis suppressor, NDRG1, in adult cancers, it is demonstrated herein for the first time that they induce NDRG1 in all three pediatric tumor cell-types, validating its role as a potential target. In fact, siRNA studies indicated that NDRG1 was responsible for MGMT down-regulation that may prevent TMZ resistance. Examining the effects of combining thiosemicarbazones with CX, ETO, or TMZ, the most promising synergism was obtained using CX. Of interest, a positive relationship was observed between NDRG1 expression of the cell-type and the synergistic activity observed in the combination of thiosemicarbazones and CX. These studies identify novel thiosemicarbazone targets relevant to childhood cancer combination chemotherapy.

## 1. Introduction

As a result of therapeutic advances in pediatric oncology, almost 83% of patients survive long term [1]. Nevertheless, aggressive treatments used for tumors in children may result in several off-target effects, including increased risks of subsequent neoplasms and serious cardiomyopathies [2,3]. Therefore, it is critical to develop effective combinatory therapeutic regimens with lower toxicity.

The clinical protocol combined oral metronomic, bio-differentiating, anti-angiogenic treatment (COMBAT) is a low-toxicity regimen for the treatment of childhood cancers and was originally designed for pediatric patients with relapsed and/or high-risk solid tumors [4]. The therapy is based on a combination of low doses of anti-angiogenic (celecoxib (CX)) and cytotoxic (etoposide (ETO) and temozolomide (TMZ)) agents with differentiation inducers (retinoids, calcitriol and its derivatives) that are administered in a metronomic regimen [5]. In general, this treatment is well tolerated and has a low acute toxicity profile [5].

The aim of the current investigation was to: (1) determine novel molecular targets of two thiosemicarbazones of the di-2-pyridylketone (DpT) class [6,7] that could be important for combinatory studies with standard chemotherapies in the COMBAT protocol for pediatric cancers; and (2) to analyze the synergy between selected chemotherapeutics used in the COMBAT protocol (CX, ETO and TMZ) and these thiosemicarbazones. The DpT class of agents has been carefully optimized over a 10-year period of structure–activity relationship analysis. These compounds were derived from aroylhydrazone ligands [8,9] and are superior to other classical thiosemicarbazones such as Triapine^®^ [6,7].

The DpT class of agents has been demonstrated by several international laboratories to possess potent and selective anti-tumor activity against a broad range of tumors in vitro and in vivo, with the compounds also being able to overcome P-glycoprotein-mediated resistance [6,7,10,11,12,13,14]. The two lead DpT agents of this class are di-2-pyridylketone-4-cyclohexyl-4-methyl-3-thiosemicarbazone (DpC) and the well-characterized parent analog, di-2-pyridylketone-4,4-dimethyl-3-thiosemicarbazone (Dp44mT) [6,7,10,11,12,13,14,15,16].

The molecular mechanism of action of DpT thiosemicarbazones involves the chelation of iron and copper within cancer cells, which is critical for proliferation and the induction of the expression of the potent metastasis suppressor N-myc downstream regulated gene-1 (NDRG1) [15,17]. These agents have been demonstrated to be effective even at very low concentrations in vitro and low doses in vivo [16,18].

The important properties of DpC and Dp44mT are their appropriate lipophilic balance, which allows facile permeation of cell membranes, and their lysosomotropic character, which results in their ability to overcome P-glycoprotein-mediated drug resistance [6,19,20,21]. Dp44mT binds iron and copper in lysosomes to form redox-active complexes that generate cytotoxic reactive oxygen species [18,19,20], which leads to permeabilization of the lysosomal membrane and the induction of apoptosis [19,21].

Moreover, many studies have demonstrated that these agents can down-regulate the key oncogenic tyrosine kinases EGFR, HER2, HER3 [22,23], and c-Met [24]. Both DpC and Dp44mT markedly suppress the activity of a variety of pro-oncogenic signaling pathways downstream of these tyrosine kinases, including the AKT, PI3K and RAS pathways [25], as well as other pathways, including the STAT3 [26], TGF-β [25,27,28], Wnt [10,27,29] and autophagic pathways [30]. As such, they subsequently inhibit proliferation, migration, the epithelial-mesenchymal transition [27], and metastasis in vivo [10].

Significantly, these novel thiosemicarbazones also synergistically increase the anti-neoplastic activity of several chemotherapeutic drugs, including gemcitabine or cisplatin [16], doxorubicin [21], paclitaxel, 5-fluorouracyl, methotrexate, 4-hydroper-oxycyclophosphamide and tamoxifen [31,32].

Considering the exciting anti-oncogenic properties of DpC and Dp44mT, three chemotherapeutics included in the COMBAT protocol, namely, CX, TMZ and ETO, were tested for their possible synergistic effects with these thiosemicarbazones.

The non-steroidal anti-inflammatory drug, CX, acts as a selective low-molecular-weight inhibitor of the heme-containing enzyme, cyclooxygenase-2 (COX-2) [33]. TMZ is a lipophilic alkylating agent that is hydrolyzed at neutral and alkaline pH values to the 5-(3-methyltriazen-1-yl)imidazole-4-carboxamide intermediate [34]. This active compound then methylates DNA bases, and thus introduces O6-methylguanine (O6-meG)/cytosine (O6-meG:C) pairs, which can result in DNA mismatches after the first round of replication [34].

Of note, ETO is often used at low doses as part of combination therapies because of its myelotoxic and leukemogenic actions, which limit its use at sufficiently high doses [35]. The primary cellular target of ETO is topoisomerase II alpha (TOP2α), which helps DNA maintain its appropriate conformation during semi-conservative DNA replication, transcription, recombination, and chromosome condensation and decondensation [36].

In this investigation, we discovered novel molecular targets of Dp44mT and DpC that could be important in terms of synergy with standard chemotherapeutics in the COMBAT protocol for pediatric tumors (CX, ETO and TMZ). This was done to better define the conditions necessary to rationally achieve synergy and develop safe and effective drug combinations. Studies were also performed to analyze for synergy between the thiosemicarbazones and these agents. These results demonstrate several novel molecular targets of thiosemicarbazones in pediatric tumor cells and identifies promising synergistic interactions particularly with CX.

## 2. Results

### 2.1. Synergism between Thiosemicarbazones and Established Chemotherapeutics Is Marked with CX

Our initial studies examined the anti-proliferative activity of the selected drugs and the interactions between thiosemicarbazones and the selected chemotherapeutics in SH-SY5Y neuroblastoma, Daoy medulloblastoma and Saos-2 osteosarcoma cells. The interactions of the tested drugs were evaluated in terms of changes in cellular proliferation after a 72-h incubation period. From these data, the concentration at which cellular proliferation was reduced by half (IC_50_; Table 1) and combination index (CI; Table 2) values were calculated.

Generally, based on the IC_50_ values identified for all three cell-types, both the thiosemicarbazones, DpC and Dp44mT, demonstrated potent activity (0.8 nM–9.3 nM; Table 1). The anti-proliferative activity of each of these compounds was markedly and significantly (*p* < 0.001) greater than that of CX (73.7–90.6 μM), TMZ (112.4–212.9 μM) and ETO (2.8–11.5 μM; Table 1). Of all the agents tested, Dp44mT demonstrated on average the greatest anti-proliferative activity in all three cell-types, while TMZ was the least effective. The sensitivity of all cell-types to CX was similar, while SH-SY-5Y cells were consistently the most sensitive to the anti-proliferative activity of all agents (Table 1).

CI analysis revealed synergistic interactions between CX and either DpC or Dp44mT in all cell-types, except the combination of DpC and CX in SH-SY5Y cells, which was antagonistic (Table 2). Of note, the strongest synergistic interaction (i.e., strong synergy) observed in this study was between CX and DpC in Saos-2 cells. Synergism was observed for the combination of TMZ and DpC in Saos-2 cells (Table 2). Slight synergy was detected for TMZ and DpC in Daoy cells, while Dp44mT and TMZ had antagonistic effects in these cells (Table 2). Antagonism and moderate antagonism were observed when TMZ was used in combination with DpC and Dp44mT, respectively, in SH-SY5Y cells (Table 2). A nearly additive effect was observed with the combination of TMZ and Dp44mT in Saos-2 cells. A synergistic effect was observed when either thiosemicarbazone was combined with ETO in Daoy cells (Table 2). On the other hand, incubation of Saos-2 or SH-SY5Y cells with either thiosemicarbazone or ETO induced antagonistic effects (Table 2).

Due to the differential effects observed in the selected cell-types and for the different combinations of drugs (Table 2); in the next part of the study, we examined the molecular mechanisms of the interactions between the thiosemicarbazones and chemotherapeutics.

### 2.2. DpC and Dp44mT Up-Regulate COX-2 Expression

**Figure 1:**
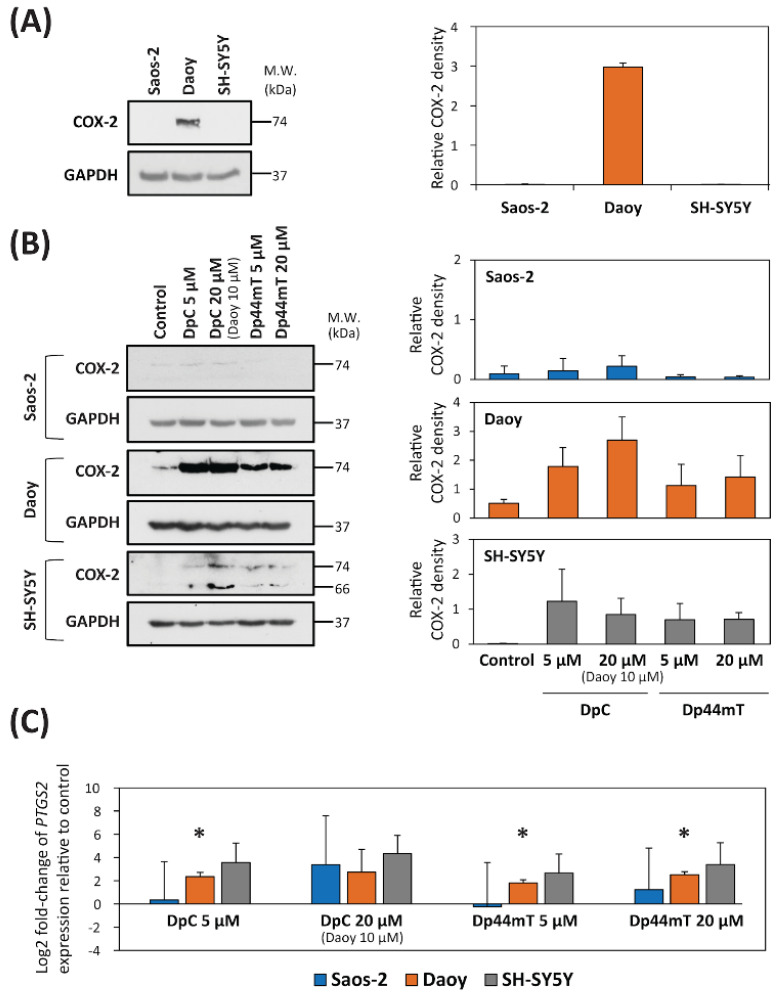
COX-2 expression is up-regulated at the (**A**,**B**) protein and (**C**) mRNA levels after incubation with DpC or Dp44mT for 24 h. (**A**) Western blot analysis of endogenous COX-2 levels in untreated (control) cells. GAPDH served as a protein loading control. (**B**) Western blot analysis of COX-2 levels in Saos-2, Daoy and SH-SY5Y cells after 24 h of incubation with either control medium, DpC (5 or 20 µM; 10 µM for Daoy cells—due to cytotoxicity) or Dp44mT (5 or 20 µM). GAPDH served as a loading control. (**C**) The graph shows changes in the mRNA expression of *PTGS2* in Saos-2, Daoy, or SH-SY5Y cells after 24 h of incubation with DpC or Dp44mT using the concentrations in (**B**). These data were obtained by RT-qPCR. The levels of mRNA expression after thiosemicarbazone treatment are presented as the log_2_ fold change in mRNA levels relative to that of the untreated controls. *GAPDH* served as a reference control. For all experiments, expression was assessed in at least three biological replicates. Western blots are typical of three experiments, while the quantitation of the blots is presented as mean ± SD (3 experiments). These data were analyzed using an unpaired Welch’s *t*-test followed by the Games-Howell post hoc test. * *p* < 0.05 indicates significant differences compared to the respective control group.

The studies described above demonstrated that the combination of CX with either thiosemicarbazone resulted in a mostly synergistic interactions in all three cell-types (Table 2). Considering that COX-2 activity is a primary target of CX [33], we hypothesized that the synergy observed between CX and the thiosemicarbazones may have been due to the ability of the latter to deplete cells of iron [6,16]. Iron is essential for the biosynthesis of the heme prosthetic group of COX-2, which is critical for its enzymatic activity [37]. Iron is also required for the prosthetic groups of other proteins, and once incorporated, is known to increase protein stability [38,39]. Thus, thiosemicarbazone-mediated iron depletion could decrease COX-2 protein levels, and this effect could potentially synergize with the inhibitory effect of CX on COX-2. To examine whether the thiosemicarbazones affected COX-2 expression, immunoblotting studies were performed to first assess their effect on endogenous COX-2 protein levels in all three cell-types (Figure 1).

Under control conditions, COX-2 expression was demonstrated to be pronounced in Daoy cells, where it was identified as a single 74-kDa band, while its expression was negligible in both Saos-2 and SH-SY5Y cells (Figure 1A; Figure S1A). The effect of the thiosemicarbazones on COX-2 expression in each cell-type was then examined after a 24 h incubation of cells with either control medium, DpC (5 or 20 µM; 10 µM for Daoy cells—due to cytotoxicity), or Dp44mT (5 or 20 µM). As shown in Figure 1A, examining Saos-2 cells, COX-2 expression was negligible and was not markedly affected by the thiosemicarbazones (Figure 1B, Figure S1B). On the other hand, examining both Daoy and SH-SY5Y cells, there was a marked increase in COX-2 expression in cells incubated with the thiosemicarbazones (especially DpC) relative to the control cells (Figure 1B, Figure S1B). Examining SH-SY5Y cells, incubation with thiosemicarbazones resulted in up-regulation of both the 74-kDa band and another 66-kDa band relative to the control, while again COX-2 expression in the control was negligible. This alteration suggested that incubation with the thiosemicarbazones changed the metabolic processing of COX-2 in SH-SY5Y cells. Interestingly, it has been reported that under other experimental conditions, COX-2 can be demonstrated as a 72-kDa *N*-glycosylated protein, as well as an unglycosylated form of the protein at 66 kDa [40].

Considering that COX-2 protein expression was up-regulated in both Daoy and SH-SY5Y cells after incubation with the thiosemicarbazones, alterations in the mRNA level of *PTGS2* (the gene encoding COX-2) were also investigated (Figure 1C). In Saos-2 cells, both thiosemicarbazones had little effect on its expression at a concentration of 5 µM relative to the control, while *PTGS2* mRNA expression was slightly, but not significantly increased at a thiosemicarbazone concentration of 20 µM (Figure 1C). In contrast, assessing Daoy cells, incubation with 5 µM DpC, or 5 and 20 µM Dp44mT, resulted in a significant (*p* < 0.05) increase in *PTGS2* mRNA relative to the control (Figure 1C). Examining SH-SY5Y cells, both DpC and Dp44mT caused up-regulation of *PTGS2* mRNA, with DpC being slightly more effective than Dp44mT (Figure 1C).

Collectively, these results in Figure 1 suggest the observed synergy in the three cell-types was not based on the ability of thiosemicarbazones to influence COX-2 expression, because the strongest synergistic effect was observed in COX-2-negative Saos-2 cells. Therefore, we focused on another common target of thiosemicarbazones and CX, namely protein kinase B (AKT) [41]. It was reported that CX suppresses tumor growth without apparent involvement of COX-2 via inhibition of PI3K/AKT signaling [41]. The PI3K/AKT signaling pathway is also known to be inhibited by the metastasis suppressor, NDRG1 [25], the expression of which is up-regulated by thiosemicarbazones [15,17,27].

**Figure 2:**
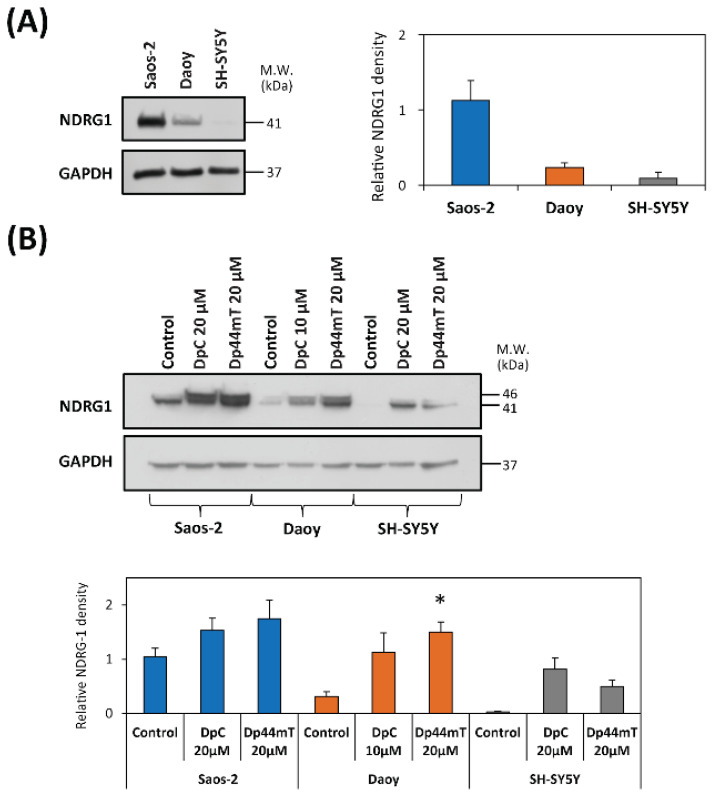
NDRG1 expression is markedly up-regulated relative to the control after a 24 h/37 °C incubation with DpC or Dp44mT. (**A**) Western blot analysis of endogenous NDRG1 levels in untreated cells. GAPDH served as a loading control. (**B**) Western blot analysis of NDRG1 levels in Saos-2, Daoy and SH-SY5Y cells after 24 h/37 °C of incubation with either control medium, DpC (20 µM; 10 µM for Daoy), or Dp44mT (20 µM). GAPDH served as a protein loading control. All experiments were repeated at least three times. Western blots are typical of three experiments, while the quantitation of the blots is presented as mean ± SD (3 experiments). These data were analyzed by unpaired Welch’s *t*-test followed by the Games-Howell post hoc test. * *p* < 0.05 indicates significant differences compared to the respective control group.

To first test the influence of thiosemicarbazones on NDRG1 expression, endogenous NDRG1 protein levels were assessed in all three cell-types (Figure 2A). Under control conditions, the highest NDRG1 levels were demonstrated in Saos-2 cells, while NDRG1 levels in Daoy cells were significantly (*p* < 0.05) lower than those in Saos-2 cells (Figure 2A; Figure S2A). Assessing SH-SY5Y cells, NDRG1 expression under control conditions was almost undetectable by immunoblotting (Figure 2A; Figure S2A). Subsequently, studies then analyzed NDRG1 expression after a 24 h incubation of cells with either control medium, Dp44mT (20 µM), or DpC (20 µM; 10 µM for Daoy cells). These data confirmed that NDRG1 was markedly up-regulated by both DpC and Dp44mT in all tested cell-types, resulting in two closely migrating bands at 41- and 46-kDa (Figure 2B, Figure S2B), as reported previously [42,43].

**Figure 3:**
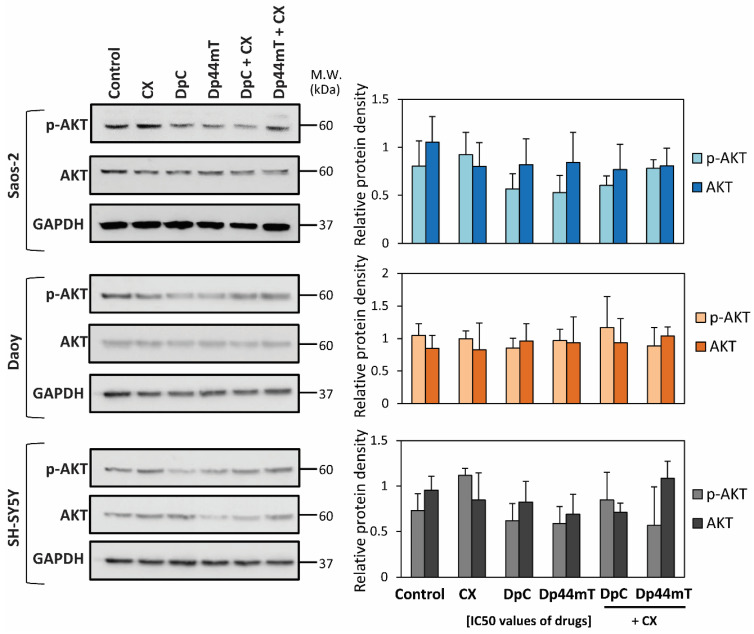
Changes in p-AKT (Ser473) level and total AKT protein expression after a 2 h/37 °C incubation of DpC, Dp44mT, CX alone, or the combinations of DpC and CX, or Dp44mT and CX (concentrations at IC_50_ levels; Table 1). Western blot analysis of p-AKT and total AKT using Saos-2, Daoy or SH-SY5Y cells incubated with control medium or this medium containing CX, thiosemicarbazones, or thiosemicarbazones combined with CX. GAPDH served as a loading control. The experiments were repeated at least three times with quantitation being the results. Western blots are typical of three experiments, while the quantitation of the blots is presented as mean ± SD (3 experiments). These data were analyzed using an unpaired Welch’s *t*-test followed by the Games-Howell post hoc test.

Next, studies focused directly on p-AKT (Ser473) levels and total AKT protein expression in the three cell-types (Figure 3, Figure S3) incubated after 2 h of treatment with IC_50_ doses of CX, DpC, Dp44mT alone or combinations of DpC or Dp44mT and CX at their IC_50_ doses (Figure 3, Figure S3). The immunoblotting analyses demonstrated that CX alone resulted in a slight increase in the p-AKT levels in Saos-2 and SH-SY5Y cells relative to the control (Figure 3, Figure S3). In contrast, p-AKT levels were slightly decreased in all tested cell-types after treatment with the thiosemicarbazones alone relative to the respective controls (Figure 3, Figure S3). In fact, examining Saos-2 cells, the p-AKT level was reduced to 65% and 70% of the control level after incubation with Dp44mT and DpC, respectively (Figure 3, Figure S3). Assessing SH-SY5Y and Daoy cells, p-AKT levels were inhibited by only approximately 10% relative to the control by thiosemicarbazones (Figure 3). Combinations of CX and the thiosemicarbazones did not have a greater effect on p-AKT and AKT levels than the agents alone (Figure 3, Figure S3).

Total AKT levels in all three cell-types were either generally slightly decreased, or not altered relative to the control level after treatment with either CX, DpC, or Dp44mT alone (Figure 3, Figure S3). The combinations of CX and the thiosemicarbazones did not lead to any substantial alterations in total AKT levels relative to the control (Figure 3, Figure S3).

Collectively, these data in Figure 3 suggested that although both thiosemicarbazones alone were able to slightly decrease AKT phosphorylation, the combination treatments were no more effective than CX or the thiosemicarbazones alone.

### 2.3. DpC and Dp44mT Down-Regulate MGMT Expression

**Figure 4:**
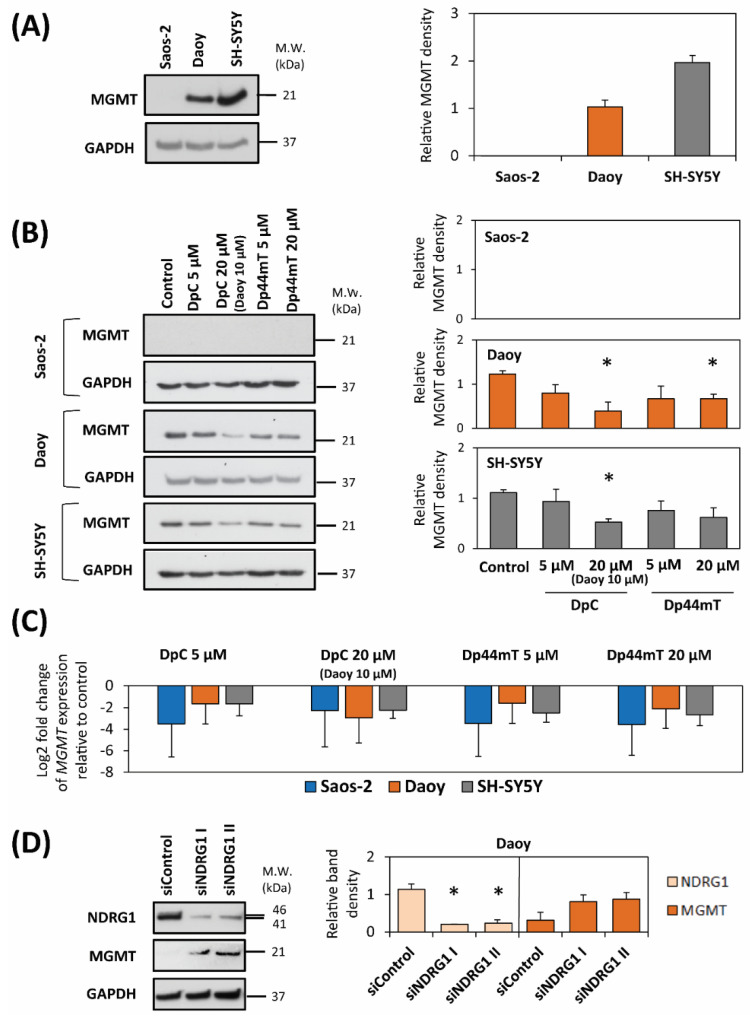
*MGMT* mRNA and protein expression was markedly down-regulated in cells after incubation with either control medium, DpC (20 µM; 10 µM for Daoy), or Dp44mT (20 µM) for 24 h/37 °C. (**A**) Western blot analysis of the endogenous MGMT level in untreated control cells. GAPDH served as a loading control. (**B**) Western blot analysis of the MGMT level in Saos-2, Daoy and SH-SY5Y cells after a 24 h/37 °C of incubation with DpC or Dp44mT. GAPDH served as a loading control. (**C**) The graph shows changes in mRNA expression of *MGMT* in Saos-2, Daoy and SH-SY5Y cells after 24 h of incubation with DpC or Dp44mT. These data were obtained by RT-qPCR. The levels of gene expression after thiosemicarbazone treatment are presented as the log_2_ fold change in mRNA expression relative to the untreated control. *GAPDH* served as a reference control. (**D**) Western blot analysis of NDRG1 and MGMT levels after *NDRG1* silencing. Two specific siRNAs for *NDRG1* were transiently transfected into Daoy cells with subsequent 48 h incubation. The cells were then transfected with a non-targeting, negative control siRNA as a relevant control. GAPDH served as a protein loading control. The western blots presented are from a typical experiment of three performed, while the quantitation of the blots is presented as mean ± SD (three experiments). These data were analyzed by the unpaired Welch’s *t*-test followed by the Games-Howell post hoc test. * (*p* < 0.05) indicates significant differences compared to the respective control group.

It is well known that the DNA repair enzyme, MGMT, inhibits TMZ activity within cells and induces resistance to TMZ therapy [44]. Therefore, to explain the differences in the anti-proliferative effects of thiosemicarbazones combined with TMZ between cell-types (see Table 2), we focused on the possible interactions between the thiosemicarbazones and MGMT expression (Figure 4). First, substantial differences in endogenous MGMT levels were found by immunoblotting between the three cell-types (Figure 4A, Figure S4A). Considering this, while no appreciable MGMT expression was detected in Saos-2 cells, SH-SY5Y cells exhibited two-fold higher MGMT levels than Daoy cells.

We then evaluated changes in MGMT levels after treatment with DpC or Dp44mT alone in the three cell-types (Figure 4B, Figure S4B). Immunoblotting revealed no appreciable MGMT expression in Saos-2 cells after treatment with thiosemicarbazones (Figure 4B, Figure S4B). This result is consistent with the almost undetectable endogenous levels of MGMT observed in this cell-type (Figure 4A, Figure S4A). On the other hand, both thiosemicarbazones alone decreased MGMT levels in Daoy and SH-SY5Y cells (Figure 4B, Figure S4B).

Reverse transcription quantitative real-time PCR (RT-qPCR) confirmed that in all three cell-types, the relative expression of the *MGMT* gene was down-regulated relative to the control after a 24 h treatment with each of the thiosemicarbazones alone at 5- or 20-µM (Daoy 10 µM DpC; Figure 4C).

To investigate the potential role of NDRG1 in the decrease of MGMT expression by thiosemicarbazones, siRNA silencing of *NDRG1* was adopted (Figure 4D, Figure S4C). For this analysis, Daoy cells were chosen as the only cell-type with detectable endogenous NDRG1 and MGMT protein levels (Figure 2A and Figure 4A). The immunoblotting revealed that the silencing of *NDRG1* by two different siRNA constructs resulted in up-regulation of MGMT levels (Figure 4D, Figure S4C). Hence, considering these results, the up-regulation of NDRG1 expression after DpC and Dp44mT treatment relative to the control (Figure 2B, Figure S2B), could lead to the down-regulation of MGMT induced by these agents, at least in Daoy cells (Figure 4B; Figure S4B).

### 2.4. DpC and Dp44mT Generally Down-Regulate the Mismatch Repair (MMR) Proteins, MSH2, MSH6 and MLH1, Which May Also Affect Cellular Sensitivity to TMZ

**Figure 5:**
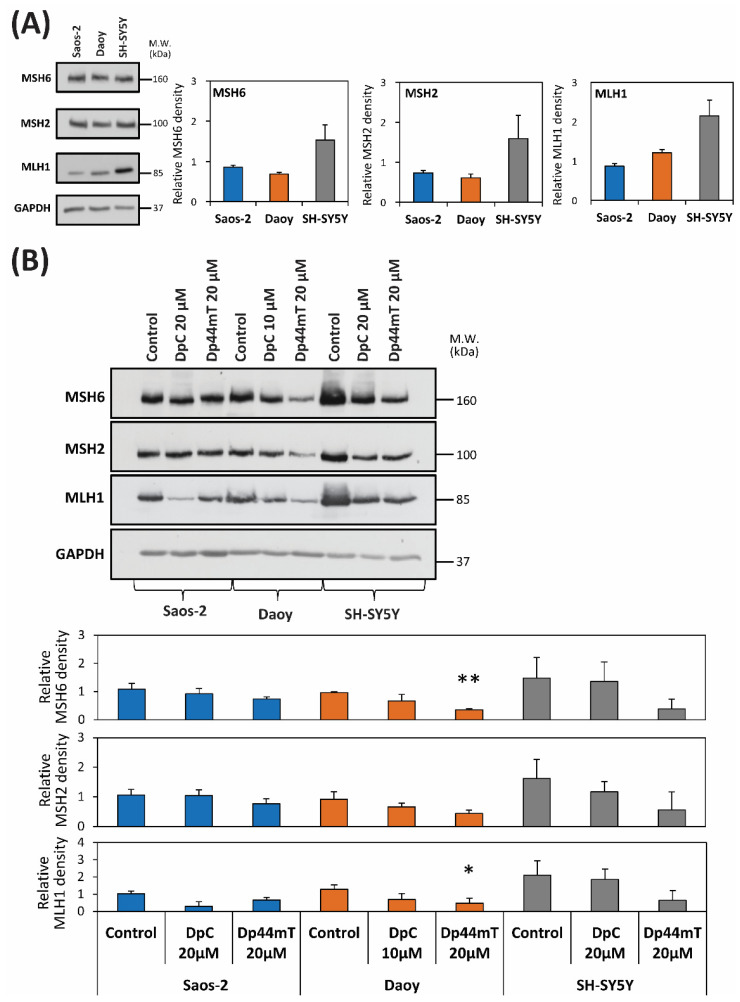
Changes in the expression of MMR proteins after incubation with either control medium, DpC (20 µM; 10 µM for Daoy), or Dp44mT (20 µM) for 24 h/37 °C. (**A**) Western blot analysis of endogenous MSH6, MSH2 and MLH1 levels in untreated cells. (**B**) MSH6, MSH2 and MLH1 levels in Saos-2, Daoy and SH-SY5Y cells after incubation with DpC or Dp44mT for 24 h. GAPDH served as a protein-loading control. The western blots presented are from a typical experiment of three performed, while the quantitation of the blots is presented as mean ± SD (3 experiments). These data were analyzed using the unpaired Welch’s *t*-test followed by the Games-Howell post hoc test. * (*p* < 0.05) and ** (*p* < 0.001) indicate significant differences compared to the respective control group.

To further understand the molecular mechanisms involved in the effects of combining TMZ with the thiosemicarbazones, studies then examined the expression of mismatch repair (MMR) proteins, which may also affect sensitivity of tumor cells to TMZ [44,45,46] (Figure 5A, Figure S5A). We compared the endogenous levels of the most important MMR proteins, namely, MSH2, MSH6, and MLH1 [47], in the three cell-types. MSH2, MSH6 and MLH1 levels were comparable in Saos-2 and Daoy cells, while for SH-SY5Y cells approximately 2-fold higher levels of each protein relative to the other two cell-types were observed (Figure 5A, Figure S5A).

Additional analyses demonstrated that a 24 h incubation with either DpC (20 µM; 10 µM for Daoy cells) or Dp44mT (20 µM), down-regulated MLH1 levels in all three cell-types (Figure 5B, Figure S5B). Decreased expression of MSH2 and MSH6 levels were also apparent in Daoy and SH-SY5Y cells after a 24 h incubation with Dp44mT or DpC, while no marked change in their expression was observed for Saos-2 cells (Figure 5B, Figure S5C). While this general decrease in MLH1, MSH2 and MSH6 after incubation with Dp44mT or DpC could be expected to aid synergistic activity with TMZ, synergism was only observed for DpC with Saos-2 and to a lesser extent with Daoy cells.

### 2.5. DpC and Dp44mT Down-Regulate TOP2α Expression

**Figure 6:**
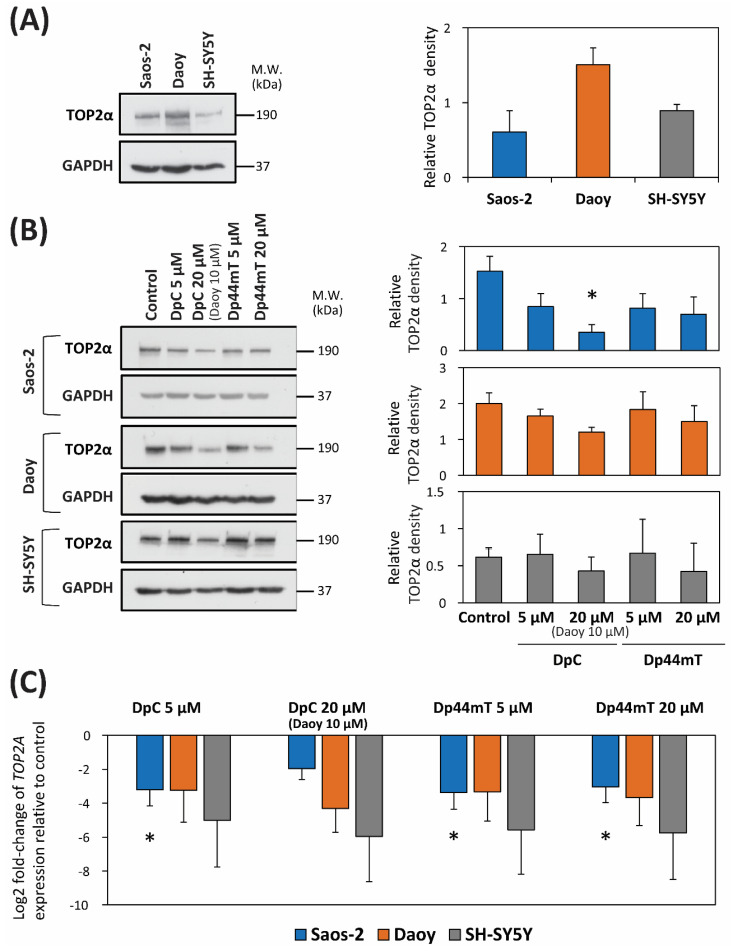
TOP2α expression is down-regulated at the (**A**,**B**) protein and (**C**) mRNA levels after incubation with either control medium, DpC (20 µM; 10 µM for Daoy), or Dp44mT (20 µM) for 24 h/37 °C. (**A**) Western blot analysis of the TOP2α level in untreated Saos-2, Daoy and SH-SY5Y cells. GAPDH served as a protein-loading control. (**B**) Western blot analysis of the TOP2α level in Saos-2, Daoy and SH-SY5Y cells after incubation with DpC or Dp44mT for 24 h. GAPDH served as a protein-loading control. (**C**) The graph shows changes in the mRNA expression of *TOP2A* in Saos-2, Daoy and SH-SY5Y cells after 24 h of incubation with DpC or Dp44mT. These data were obtained by RT-qPCR. The levels of gene expression after thiosemicarbazone treatment are presented as the log_2_ fold change in mRNA expression relative to that in the untreated control. *GAPDH* served as a reference control. For all experiments, expression was assessed in three biological replicates. The Western blots presented are from a typical experiment of three performed, while the quantitation of the blots is presented as mean ± SD (three experiments). These data were analyzed using the unpaired Welch’s *t*-test followed by the Games-Howell post hoc test. * (*p* < 0.05) indicates significant differences compared to the respective control group.

To explore the possible interaction between the thiosemicarbazones and ETO, we tested the hypothesis that DpC and Dp44mT can modulate the level of TOP2α, which is the primary target of ETO [48]. Analysis of endogenous TOP2α expression revealed that the levels of TOP2α were almost two-fold higher in Daoy cells than in SH-SY5Y and Saos-2 cells (Figure 6A, Figure S6A).

Subsequently, studies analyzed TOP2α levels after 24 h of treatment with two different concentrations of the thiosemicarbazones to determine if their effects were concentration-dependent. In general, and relative to the control, down-regulation of TOP2α levels was observed at 20 µM of either thiosemicarbazone using all three cell-types, with less effect being observed at 5 µM, especially for SH-SY5Y cells where no decrease was apparent (Figure 6B, Figure S6B). Examining *TOP2A* mRNA levels, both concentrations of the thiosemicarbazone decreased its expression in all three cell-types (Figure 6C).

## 3. Discussion

Combination therapy remains a cornerstone of cancer treatment, and as such, it is essential to investigate the synergistic activities of novel agents. The novel DpT class of thiosemicarbazones demonstrate: (1) potent and selective anti-tumor activity; (2) the ability to overcome P-glycoprotein-mediated resistance [11,21]; and (3) can inhibit metastasis via up-regulating NDRG1 [10,17,24,25,27,49,50]. In fact, our extensive previous studies, and subsequently those of others, have demonstrated that both Dp44mT [6,7,10,11,12,13,14,22,51,52] and DpC [12,15,16,22,52,53] show selective and potent anti-cancer efficacy in vitro and in vivo against a broad variety of tumor cell-types and tumors (e.g., lung cancer, melanoma, neuroblastoma, neuroepithelioma ovarian carcinoma) [6,7,10,11,12,13,14,15,16,17,18,19,20,21,22,23,24,25,26,27,28,29,30,31,32]. Due to the marked and selective activity and excellent safety and tolerability of DpC, the agent was examined in multi-centre, Phase I clinical trials in humans [54].

As a pertinent example of their utility in pediatric oncology, our previous studies in vitro have demonstrated that both DpC and Dp44mT (at 2.5 μM) showed no pronounced anti-proliferative activity against the non-tumorigenic, immortalized cell lines (i.e., MSC, H9C2, MIHA, and HK2), but demonstrated marked anti-tumor efficacy against neuroblastoma cells [12]. This led to studies using orthotopic neuroblastoma xenografts, which demonstrated that DpC significantly inhibited tumor growth and was well tolerated [12]. Considering these results collectively, which demonstrate potent and safe anti-cancer activity, it was important to investigate the ability of these thiosemicarbazones to synergize with established chemotherapies for the treatment of childhood cancers.

As shown in the current investigation, both thiosemicarbazones (DpC and Dp44mT) potentiated the cytotoxic activity of CX in particular, and to a much lesser extent, TMZ and ETO (Table 2). The synergistic interactions of the thiosemicarbazones with each chemotherapeutic drug assessed probably resulted from different pharmacological interactions.

### 3.1. Combination of the Novel Thiosemicarbazones and CX

The combination of DpC or Dp44mT and CX exhibited enhanced anti-proliferative activity and acted generally synergistically in all three cell-types, except for DpC treated SH-SY5Y cells. We hypothesized that COX-2 activity can be modulated by both CX and thiosemicarbazones, as the catalytic domain of CX contains an iron-dependent heme prosthetic group that is necessary for its activity [55]. Due to the well-known iron chelation activity of DpC and Dp44mT [6,16], it can be hypothesized that thiosemicarbazones chelate cellular iron necessary for heme biosynthesis, which is critical for COX-2 function.

To examine the effect of DpC or Dp44mT on COX-2 expression, their effects were assessed using Western blot analysis and qRT-PCR, with these studies demonstrating up-regulation of COX-2 expression at the mRNA and protein levels in Daoy and SH-SY5Y, while Saos2 cells did not express appreciable COX-2 protein levels. The observed increase in COX-2 expression after incubation with thiosemicarbazones can be speculated to be a “rescue” attempt by the cells to compensate for the loss of COX-2 function.

Assessing the mechanism of synergy between the thiosemicarbazones and CX, it is of interest that the strongest synergy for this combination was observed in COX-2 negative Saos-2 cells (Table 2). These data indicated that the mechanism of synergy in this cell-type was independent of COX-2 and suggested the existence of another molecular target. Other molecular targets of CX include AKT, its upstream kinase 3-phosphoinositide-dependent kinase-1 [41], cyclin-dependent kinase inhibitors and cyclins [52], the anti-apoptotic proteins survivin, Bcl-2 and Mcl-1 [56], and the sarcoplasmic/endoplasmic reticulum calcium ATPase [57]. Considering this, thiosemicarbazones have been demonstrated to target AKT and cyclin-dependent kinase inhibitors, e.g., p21 and p27, with many of their effects being mediated by the up-regulation of the metastasis suppressor, NDRG1 [25,51,58,59,60].

As such, to examine whether NDRG1 could be involved in the synergistic activity of thiosemicarbazones with CX (Table 2), the expression of endogenous NDRG1 was examined. These studies demonstrated that NDRG1 expression was up-regulated in all three cell-types by both thiosemicarbazones. Examining Saos-2 cells, which exhibited the highest NDRG1 expression (Figure 2A,B), it was demonstrated that both thiosemicarbazones exhibited strong synergy with CX. Assessing SH-SY5Y cells, which expressed the lowest NDRG1 level, Dp44mT and CX exhibited only moderate synergy, while DpC and CX had an antagonistic effect. Thus, it can be suggested that the synergistic activity of the thiosemicarbazones and CX could be associated with high endogenous NDRG1 levels.

### 3.2. Combination of Novel Thiosemicarbazones and TMZ

A synergistic effect of the novel thiosemicarbazones in combination with TMZ was only observed for DpC-treated Saos-2 cells, with slight synergism being identified in Daoy cells (Table 2). In the other cell-types the combination of thiosemicarbazones and TMZ had additive or antagonistic effects. Given the mechanism of action of TMZ, we hypothesized that the observed differences in the effects of the different drug combinations among the cell-types could be due to different expression of either: (1) MGMT, whose repair activity inhibits TMZ activity and induces resistance to this drug [61,62]; or (2) proteins belonging to the MMR system (MSH2, MSH6, and MLH1) of DNA repair, which may also influence to TMZ sensitivity [63]. Of note, it has been demonstrated that binding of Zn^2+^ is necessary for the proper function of MGMT [64]. As DpC and Dp44mT chelate Zn(II) [20], we hypothesized that DpC and Dp44mT decrease MGMT activity by this mechanism and may potentiate TMZ anti-tumor activity.

Our results demonstrated that thiosemicarbazones down-regulate MGMT protein expression in all tested cell-types except Saos-2 cells, which did not express appreciable MGMT protein levels (Figure 4A,B). As the strongest synergism was observed in Saos-2 cells incubated with DpC (Table 2), it was unlikely that MGMT expression was a major molecular target that led to this response. Additionally, while thiosemicarbazones down-regulate MGMT in Daoy and SH-SY5Y cells, antagonism or slight synergism was observed for the combination of thiosemicarbazones and TMZ (Table 2). Again, this indicated MGMT down-regulation by thiosemicarbazones did not lead to marked synergism.

Because MGMT is a significant molecular target, and since the thiosemicarbazones down-regulate its protein levels in Daoy and SH-SY5Y cells, it was of interest to understand the mechanism of this activity, which could be beneficial for understanding the efficacy of these agents. One of the important effectors of DpC and Dp44mT anti-tumor activity is NDRG1 expression [7,10,15,17,22,23,25,27,28,29]. It is well known that thiosemicarbazones and NDRG1 can inhibit Wnt signaling and effects [10,27,29], with inhibition of Wnt activity being reported to down-regulate MGMT expression that restored chemosensitivity to TMZ [65].

Based on the relationship between the marked increase in NDRG1 expression (Figure 2B) and decreased MGMT protein levels after thiosemicarbazone treatment (Figure 4B), we hypothesized that NDRG1 up-regulation by thiosemicarbazones down-regulated MGMT. This premise was confirmed by *NDRG1* silencing using two different siRNA constructs, where inhibition of NDRG1 expression resulted in increased MGMT levels in Daoy cells (Figure 4D).

NDRG1 is a metastasis suppressor that acts through an impressive array of anti-oncogenic effectors in addition to MGMT [7,10,15,17,22,23,25,27,28,29]. As such, the ability of thiosemicarbazones to up-regulate NDRG1 may relate to the synergistic activity observed between TMZ and DpC in Saos2 and Daoy cells, although further studies are essential to demonstrate this. From these studies, and also the correlation between NDRG1 expression and synergism with CX discussed above, it can be concluded than NDRG1 up-regulation by thiosemicarbazones is an important factor for their synergistic activity upon combination with other drugs in pediatric tumor cell-types.

The proteins MSH6, MSH2 and MLH1 are necessary for the coordinated, multi-step process of excision and replacement of nucleic acid bases to ensure DNA repair [66]. Considering that the thiosemicarbazones down-regulated MMR protein expression particularly in Daoy and SH-SY5Y cells (Figure 5B), this would presumably aid the DNA alkylating activity of TMZ, and may explain the synergistic or additive interactions of thiosemicarbazones with TMZ in Saos-2 and Daoy cells (Table 2). However, the general down-regulation of MMR expression by the thiosemicarbazones does not explain the antagonism or moderate antagonism observed after their combination with TMZ in other cell-types. These data suggest more complex interactions that need to be further assessed.

### 3.3. Combination of Novel Thiosemicarbazones and ETO

The combination of DpC or Dp44mT and ETO only had synergistic effects in Daoy cells, whereas for SH-SY5Y and Saos-2 cells, antagonism was observed. When analyzing the possible drug interactions, we focused on the fact that the thiosemicarbazone, Dp44mT, and ETO inhibit the activity of TOP2α [36,67]. However, while Dp44mT was reported in one study to inhibit TOP2α [67], a subsequent investigation did not confirm this result and demonstrated Dp44mT did not affect TOP2α activity [68]. For both these studies by others, the effect of neither DpC nor Dp44mT was assessed directly on the basis of mRNA or protein levels of TOP2α. In contrast, the current investigation demonstrated DpC and Dp44mT down-regulated TOP2α mRNA and protein expression of TOP2α in all three cell-types.

Based on these latter results, we expected synergism between thiosemicarbazones and ETO. This was predicted because ETO is a TOP2α poison, which is well known to inhibit tumor cell growth [36,69]. Thus, having two pharmacologically different types of agents (thiosemicarbazones and ETO) acting on the same molecular target by different mechanisms (i.e., expression and enzymatic activity) would potentially be beneficial, leading to synergism. On the other hand, previous studies have demonstrated that an important determinant of sensitivity to TOP2α poisons is the overall endogenous level of TOP2α, with low TOP2 levels showing resistance to ETO [69,70]. As such, antagonism between thiosemicarbazones that decrease TOP2α expression and ETO could also be theoretically envisioned.

The Daoy cell-type expressed the highest endogenous level of TOP2α of the three cell-types and demonstrated synergistic activity with the combination of the thiosemicarbazones and ETO. In contrast, lower levels of TOP2α were observed in Saos-2 and SH-SY5Y cells where antagonism was identified. In all cell types, there was a decrease in TOP2α expression after incubation with either of the thiosemicarbazones. Considering these facts, it can be speculated that the relative levels of TOP2α could be important in terms of whether synergism or antagonism is observed. However, further studies are required to definitely determine the molecular mechanism involved.

In summary, the collective results of this investigation identified novel molecular targets of thiosemicarbazones in pediatric cancer cell-types. Moreover, the work provides new mechanistic insight into the alterations in anti-tumor efficacy of CX, ETO and TMZ upon combination with DpC or Dp44mT. As DpC has entered clinical trials [11], these data are useful for guiding future combinatory studies.

## 4. Materials and Methods

### 4.1. Chemicals

DpC and Dp44mT were synthesized and characterized as described previously [16,18]. Both thiosemicarbazones were prepared as stock solutions in DMSO at a concentration of 100 mM (Sigma-Aldrich, St. Louis, MO, USA) and then diluted in cell culture medium to achieve a DMSO concentration <0.5% (*v*/*v*). At this concentration of DMSO there was no effect on cellular proliferation relative to control medium [8]. The clinically used chemotherapeutics, namely ETO (Cat. No. E1383), CX (Cat. No. PZ0008) and TMZ (Cat. No. T2577), were obtained from Sigma-Aldrich, prepared as stock solutions in DMSO at a concentration of 100 mM, and then diluted as described above.

### 4.2. Cell Culture

Saos-2 osteosarcoma cells (Cat. No. HTB-85) and Daoy medulloblastoma cells (Cat. No. HTB-186™) were purchased from the American Type Culture Collection (Manassas, VA, USA). SH-SY5Y neuroblastoma cells (Cat. No. 94030304) were purchased from The European Collection of Authenticated Cell Cultures (Salisbury, UK).

The Daoy and Saos-2 cells were cultured in Dulbecco’s modified Eagle’s medium (DMEM) supplemented with 10% fetal calf serum (FCS), 2 mM glutamine, and the antibiotics, penicillin (100 IU/mL) and streptomycin (100 μg/mL) (all from GE Healthcare Europe GmbH, Freiburg, Germany). The medium used for Daoy cells was supplemented with 1% non-essential amino acids (Biosera, Nuaille, France).

SH-SY5Y cells were cultured in a mixture of DMEM/F12 (1:1) supplemented with 20% FCS, 2 mM glutamine and the antibiotics, penicillin (100 IU/mL) and streptomycin (100 μg/mL) (all from GE Healthcare), and 1% nonessential amino acids (Biosera).

All cells were maintained under standard cell culture conditions at 37 °C in an atmosphere of 95% air, 5% CO_2_.

### 4.3. siRNA

Two specific siRNAs for *NDRG1* were used, namely siNDRG1 I (Cat. No. AM16708, ID: 135611; ThermoFisher Scientific, Waltham, MA, USA) and siNDRG1 II (Cat. No. AM16708, ID 135612; ThermoFisher Scientific). These were compared with non-targeting negative control siRNA (Cat. No. 4390846, ThermoFisher Scientific). The siRNA was transiently transfected into Daoy cells using Lipofectamine RNAiMAX (ThermoFisher Scientific) and incubated for 48 h/37 °C.

### 4.4. Treatment Protocol

The 3-[4,5-dimethylthiazol-2-yl]-2,5-diphenyltetrazolium bromide (MTT) proliferation assay (see Section 4.5, below) was employed to quantify synergy between the selected drugs and thiosemicarbazones. Cells were seeded in 96-well plates at a density of 5 × 10^3^ cells/well (SH-SY5Y and Saos-2 cells) or 2 × 10^3^ cells/well (Daoy cells) in 100 µL of complete DMEM. Different seeding densities were used to ensure that the cells remained in the log phase of growth during drug incubation.

The cells were allowed to adhere overnight, and the medium was then removed and replaced with fresh medium containing the appropriate concentrations of either of the drugs (DpC, Dp44mT, CX, ETO, or TMZ) alone or in appropriate combinations. The drug concentrations used for combination treatments were based on the initial IC_50_ values (⅛-, ¼-, ½-, 1-, 2-, 4-, and 8-fold of IC_50_) of each drug, and therefore, they were specific for each cell line examined. The plates were incubated for 3 days at 37 °C.

To investigate the molecular effects of the thiosemicarbazones in combined treatments, samples were prepared for immunoblotting and RT-qPCR. Cells were seeded in Petri dishes (90 mm diameter) at a density of at 5 × 10^5^/dish and allowed to adhere overnight. The next day, the cells were treated with the individual thiosemicarbazones at concentrations higher than the IC_50_ value for 24 h at 37 °C, and the expression changes then examined.

This shorter incubation period of 24 h prevented cytotoxicity, which would confound data interpretation. Therefore, the following concentrations were chosen: DpC: 5 μM and 20 μM; Dp44mT: 5 μM and 20 μM. Of note, due to the high cytotoxicity of 20 μM DpC in Daoy cells, DpC was used in these cells at concentrations of 5 μM and 10 μM. Treated cells and untreated controls were incubated under standard conditions for 24 h/37 °C.

In addition, the effects of combination treatment with the thiosemicarbazones and CX were analyzed in more detail. For this purpose, cells plated in Petri dishes (90 mm diameter) were treated with the IC_50_ doses of DpC, Dp44mT, CX, or the combinations of DpC and CX, or Dp44mT and CX. The cells were then incubated under standard conditions for 2 h/37 °C before being processed for immunoblotting.

### 4.5. Cell Proliferation

The MTT assay was used to evaluate cell proliferation and was performed as described [71]. As shown previously, the number of cells was demonstrated to be a linear function of MTT absorbance [8]. Briefly, the cells were treated and incubated for 3 days (see Section 4.3). Subsequently, they were then incubated with MTT (0.5 mg/mL; Sigma-Aldrich) for 3 h/37 °C. Then, the formazan crystals were dissolved in 200 μL of DMSO. The absorbance was measured at 570 nm with a reference absorbance of 620 nm using a Sunrise Absorbance Reader (Tecan, Männedorf, Switzerland).

### 4.6. Calculation of CI

CIs were calculated to quantitatively compare the dose-effect relationship of each drug alone or in combination to determine whether a given combination acts synergistically. CIs were calculated from growth inhibition curves, as previously described [16]. A 1:1 ratio of drugs was used for combination treatments. The CI values were determined using CalcuSyn software (version 2.0, Biosoft, Cambridge, UK). The Chou Talalay method was adopted to identify synergism (CI < 0.9), additive effect (CI = 0.9–1.1) or antagonism (CI > 1.1) [72].

### 4.7. RT-qPCR

The relative expression of selected genes was analyzed using RT-qPCR. Total RNA was isolated and reverse transcribed into cDNA by established methods [63]. RT-qPCR was performed in a 10-μL reaction using a Kapa Biosystems Quantitative Real-Time PCR kit (Kapa Biosystems, Wilmington, MA, USA) and analyzed using a 7500 Fast Real-Time PCR System and 7500 Software v. 2.0.6 (both Life Technologies, Carlsbad, CA, USA). Changes in transcript levels were examined using Cq values normalized to the housekeeping gene, GAPDH. The primer sequences used for TOP2A, PTGS2, MGMT and the GAPDH genes are provided in Table 3.

### 4.8. Immunoblotting Assay

Protein extracts were collected using LB1 lysis buffer (50 mM Hepes-KOH, pH 7.5, 140 mM NaCl, 1 mM EDTA, 10% glycerol, 0.5% NP-40, and 0.25% Triton X-100). Total proteins (50 μg/well) were loaded onto 10% polyacrylamide gels and electrophoresed. Subsequently, the separated proteins were blotted onto PVDF membranes (Bio-Rad Laboratories, Munich, Germany).

The membranes were blocked for 1 h/20 °C with 5% BSA (Sigma-Aldrich) in PBS with 0.1% Tween-20 (Sigma-Aldrich) to detect p-AKT, or with 5% non-fat dry milk in PBS with 0.1% Tween-20 to detect all other proteins.

The blocked membranes were incubated overnight with the primary monoclonal antibodies listed in Table 4. The next day, the membranes were incubated with secondary antibodies for 1 h/20 °C (Table 4). ECL-Plus detection was carried out according to the manufacturer’s instructions (GE Healthcare, Little Chalfont, UK). Quantification of the protein bands on western blots was performed using ImageJ software (NIH, Bethesda, MD, USA).

### 4.9. Statistics

SPSS Statistics software (version 25.0, IBM, New York, NY, USA) was used for statistical analysis. Numerical data are presented as the mean ± standard deviation (of three independent experiments). Statistical comparisons of the RT-PCR and immunoblotting results were performed using an unpaired Welch’s *t*-test. *p* < 0.05 (*) indicates significant differences compared to the respective control group. In all studies, experiments were independently performed at least three times.

## 5. Conclusions

In conclusion, the current investigation clearly demonstrated that DpC and Dp44mT potentiate the cytotoxic effects of selected chemotherapeutics used in metronomic regimens in pediatric oncology. The most marked synergism of the thiosemicarbazones was observed with CX, while synergism or slight synergism was only found for TMZ with DpC in two cell-types. Etoposide demonstrated largely antagonistic effects with the thiosemicarbazones, while nearly additive activity was found for Daoy cells.

In terms of mechanistic dissection, this study discovered several novel targets of thiosemicarbazones that had not been identified previously. These new targets could be important to consider when assessing possible drug combinations. For instance, this study demonstrated that both DpC and Dp44mT caused: (1) substantial up-regulation of COX-2 protein expression, but only in Daoy and SH-SY5Y cells; (2) down-regulation of the DNA repair protein MGMT in Daoy and SH-SY5Y cells; (3) down-regulation of MMR repair proteins, MSH2 and MSH6, in Daoy and SH-SY5Y cells; and (4) down-regulation of the MMR repair protein, MLH1, and Topo-2α in all three cell-types.

Additionally, as demonstrated previously in a variety of adult cancer cell-types, the thiosemicarbazones demonstrated strong activity at up-regulating NDRG1 in all three pediatric tumor cell lines. The up-regulation of NDRG1 was responsible for the down-regulation of MGMT in Daoy cells, which is important for preventing drug resistance to agents such as TMZ. Furthermore, a positive relationship was observed between NDRG1 levels of the cell-type and the synergistic activity observed in the combination of thiosemicarbazone and CX.

Of the combinations examined, the most promising was that using CX and either DpC or Dp44mT. This may be related to the ability of the thiosemicarbazones to up-regulate COX2 expression, although further studies are required to validate this.

In conclusion, the results herein provide new insight into the alterations in anti-neoplastic activity of CX, ETO and TMZ upon combination with DpC or Dp44mT. As DpC has entered clinical trials [11], these data could be important for guiding future combinatory studies in vivo in animal models and eventually in human trials.

## Figures and Tables

**Table 1:** IC_50_ values. The IC_50_ values of the individual drugs were determined in each cell-type after incubation with each drug alone for 72 h/37 °C. The results are presented as the mean ± SD (3 experiments).

Cell Line	IC_50_ Values				
	CX [µM]	TMZ [µM]	ETO [µM]	DpC [nM]	Dp44mT [nM]
Saos-2	75.6 ± 7.1	116.5 ± 3.0	11.5 ± 0.7	8.8 ± 0.2	9.3 ± 0.6
Daoy	90.6 ± 6.7	212.9 ± 38.4	6.8 ± 2.0	8.5 ± 2.2	5.4 ± 0.8
SH-SY5Y	73.7 ± 9.1	112.4 ± 3.8	2.8 ± 0.3	3.7 ± 0.6	0.8 ± 0.1

**Table 2:** Quantitative assessment of drug interactions—combination index (CI) values. Analysis of the interaction between CX, TMZ, or ETO, and the thiosemicarbazones (DpC and Dp44mT) was performed using CalcuSyn software. The data were obtained after a 72 h/37 °C incubation with each agent alone or in combination. The CIs were calculated from growth inhibition curves. A 1:1 ratio of drugs was used for combination treatments. The Chou Talalay method was adopted to identify synergistic, additive and antagonistic activity. These results are mean ± SD (three experiments).

Cell Line	Combination Index (CI) ± SD								
	CX		TMZ				ETO		
	+DpC	+Dp44mT	+DpC			+Dp44mT	+DpC	+Dp44mT	
Saos-2	0.29 ± 0.19	0.33 ± 0.15	0.48 ± 0.07			0.92 ± 0.37	1.55 ± 0.44	2.19 ± 0.70	
Daoy	0.52 ± 0.03	0.39 ± 0.05	0.89 ± 0.11			1.59 ± 0.31	0.45 ± 0.12	0.64 ± 0.12	
SH-SY5Y	1.56 ± 0.08	0.84 ± 0.11	1.62 ± 0.32			1.38 ± 0.25	1.66 ± 0.66	1.67 ± 0.25	
**Categories of Interactions**									
0.10–0.30	strong synergism				0.91–1.10	nearly additive			
0.31–0.70	synergism				1.11–1.20	slight antagonism			
0.71–0.85	moderate synergism				1.21–1.45	moderate antagonism			
0.86–0.90	slight synergism				1.46–3.30	antagonism			

**Table 3:** Sequences of the primers used for RT-qPCR.

Gene	Primer Sequence	Product Length (bp)
*GAPDH*	F: 5′-AGC CAC ATC GCT CAG ACA CC-3′ R: 5′-GTA CTC AGC GCC AGC ATC G-3′	302
*MGMT*	F: 5′-CCGTTTGCGACTTGGTACTTG-3′ R: 5′-TGGTGAACGACTCTTGCTGG-3′	312
*PTGS2*	F: 5′-GATGATTGCCCGACTCCCTT-3′ R: 5′-TGAAAAGGCGCAGTTTACGC-3′	273
*TOP2A*	F: 5′-ACCATTGCAGCCTGTAAATGA-3′ R: 5′-GGGCGGAGCAAAATATGTTCC-3′	129

Abbreviations: F, forward; R, reverse.

**Table 4:** Primary and secondary antibodies used in this study.

**Primary Antibodies**					
**Antigen**	**Type/Host**	**Clone**	**Catalog No.**	**Manufacturer**	**Dilution**
AKT (pan)	* Mono/Rb	C67E7	4691S	CST	1:2000
p-AKT (Ser473)	Mono/Rb	D9E	4060S	CST	1:2000
COX-2	Mono/Rb	D5H5	12282S	CST	1:1000
GAPDH	Mono/Rb	14C10	2118S	CST	1:10,000
MGMT	Mono/Mo	-	51234M	Bioss	1:1000
MLH1	Mono/Mo	4C9C7	3515S	CST	1:1000
MSH2	Mono/Rb	D24B5	2017S	CST	1:2500
MSH6	Mono/Rb	D60G2	5424S	CST	1:2500
NDRG1	Mono/Rb	-	9485	CST	1:2000
TOP2α	Mono/Rb	D10G9	12286	CST	1:1000
**Secondary Antibodies**					
**Host**	**Specificity**	**Conjugate**	**Catalog No.**	**Manufacturer**	**Dilution**
Goat	Anti-Rb IgG	HRP	7074	CST	1:5000
Horse	Anti-Mo IgG	HRP	7076	CST	1:5000

* Abbreviations: Mono, monoclonal; Rb, rabbit; Mo, mouse; CST, Cell Signaling Technology Inc., Danvers, MA, USA; Bioss, Bioss Antibodies Inc., Woburn, MA, USA.

## Supplementary Materials

The following are available online at https://www.mdpi.com/2072-6694/12/12/3781/s1, Figure S1: COX-2 levels in untreated cell lines and after the treatment with thiosemicarbazones, Figure S2: NDRG1 levels in untreated cell lines and after the treatment with thiosemicarbazones, Figure S3: p-AKT and AKT levels after the treatment with IC50 doses of CX, DpC, Dp44mT and their combinations, Figure S4: MGMT levels in untreated cell lines and after the treatment with thiosemicarbazones, Figure S5: Levels of mismatch repair proteins in untreated cell lines and after the treatment with thiosemicarbazones, Figure S6: TOP2α levels in untreated cell lines and after the treatment with thiosemicarbazones.
