# Supplementary material for: Novel Thiosemicarbazones Sensitize Pediatric Solid Tumor Cell-Types to Conventional Chemotherapeutics through Multiple Molecular Mechanisms
Source: Cancers (Basel). 2020 Dec 15;12(12):3781. doi: 10.3390/cancers12123781 (PMC7765366; doi:10.3390/cancers12123781)
Supplement: Supplementary file 1 [file cancers-12-03781-s001.pdf]

SUPPLEMENT 1A

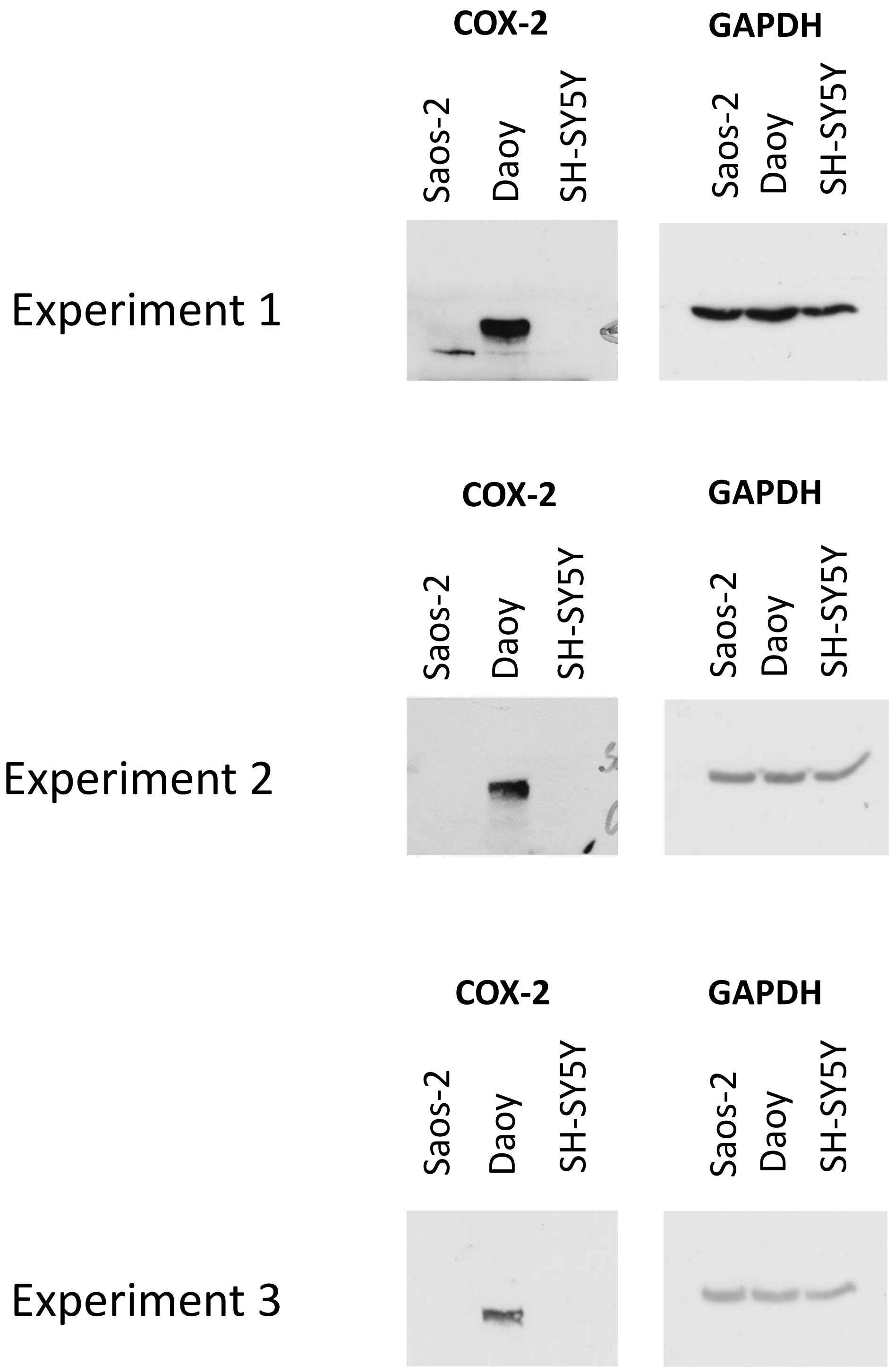

SUPPLEMENT 1A : COX-2 levels in untreated cell lines.

Western blot analysis of the endogenous COX-2 levels in untreated cell lines. GAPDH served as a loading control. The experiments were repeated three times.

SUPPLEMENT 1B

Saos-2

1 – control
2 – DpC 5 µM
3 – DpC 20 µM
4 – Dp44mT 5 µM
5 – Dp44mT 20 µM

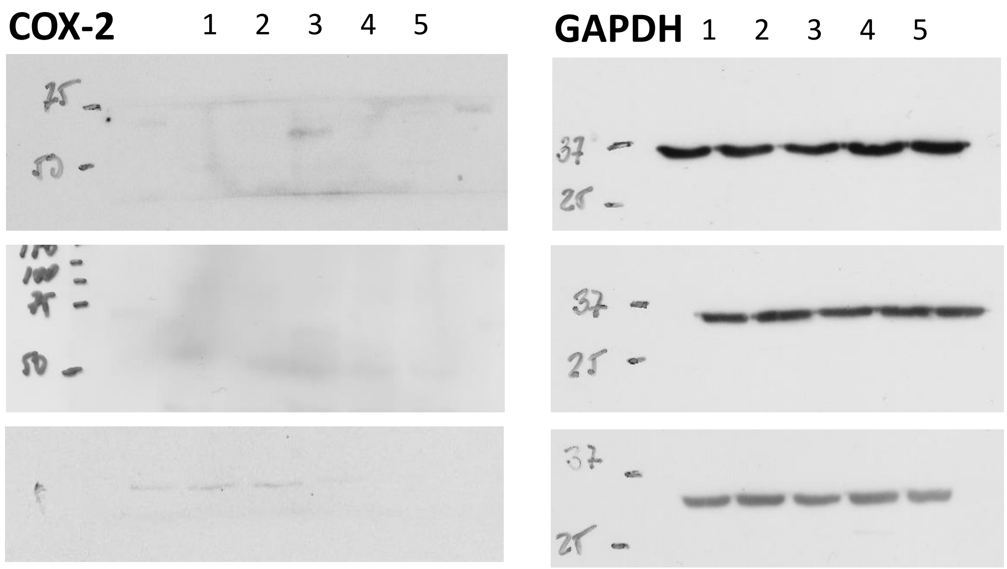

Daoy

1 – control
2 – DpC 5 µM
3 – DpC 10 µM
4 – Dp44mT 5 µM
5 – Dp44mT 20 µM

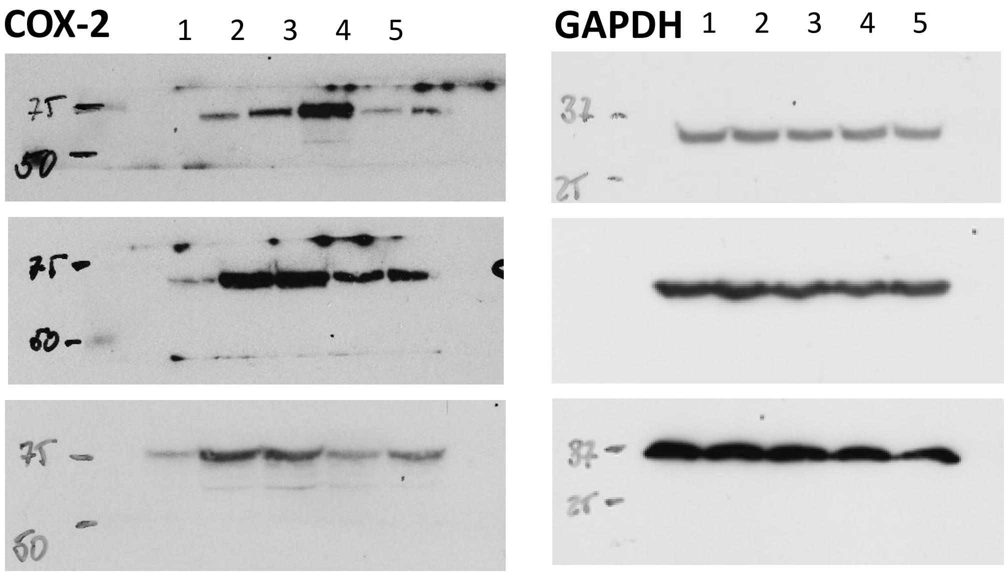

SH-SY5Y

1 – control
2 – DpC 5 µM
3 – DpC 20 µM
4 – Dp44mT 5 µM
5 – Dp44mT 20 µM

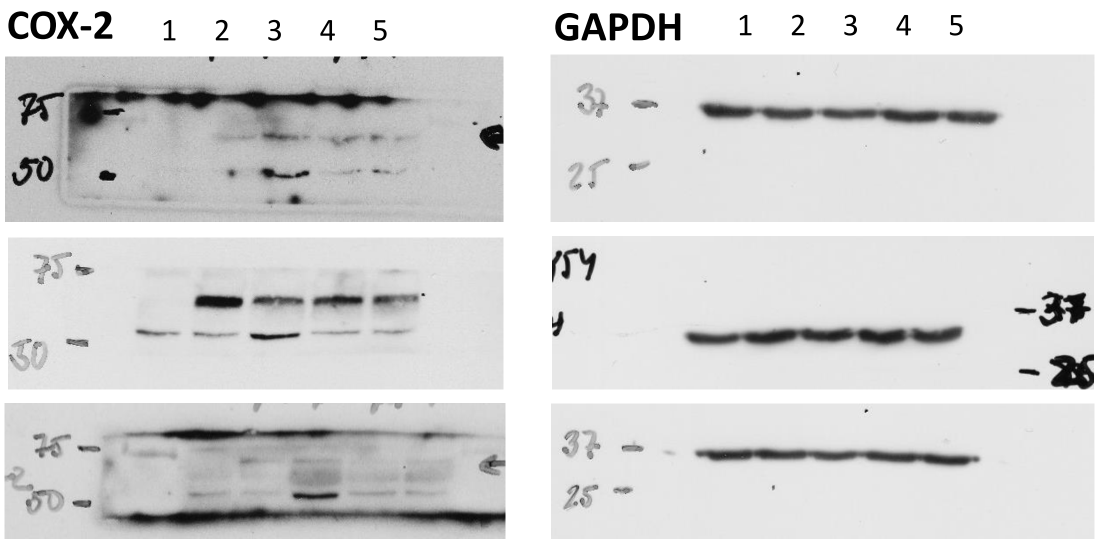

SUPPLEMENT 1B: COX-2 levels after the treatment with thiosemicarbazones.

Western blot analysis of the COX-2 levels in Saos-2, Daoy and SH-SY5Y cells after 24 h of incubation with DpC or Dp44mT. GAPDH served as a loading control. The experiments were repeated three times.

SUPPLEMENT 2A

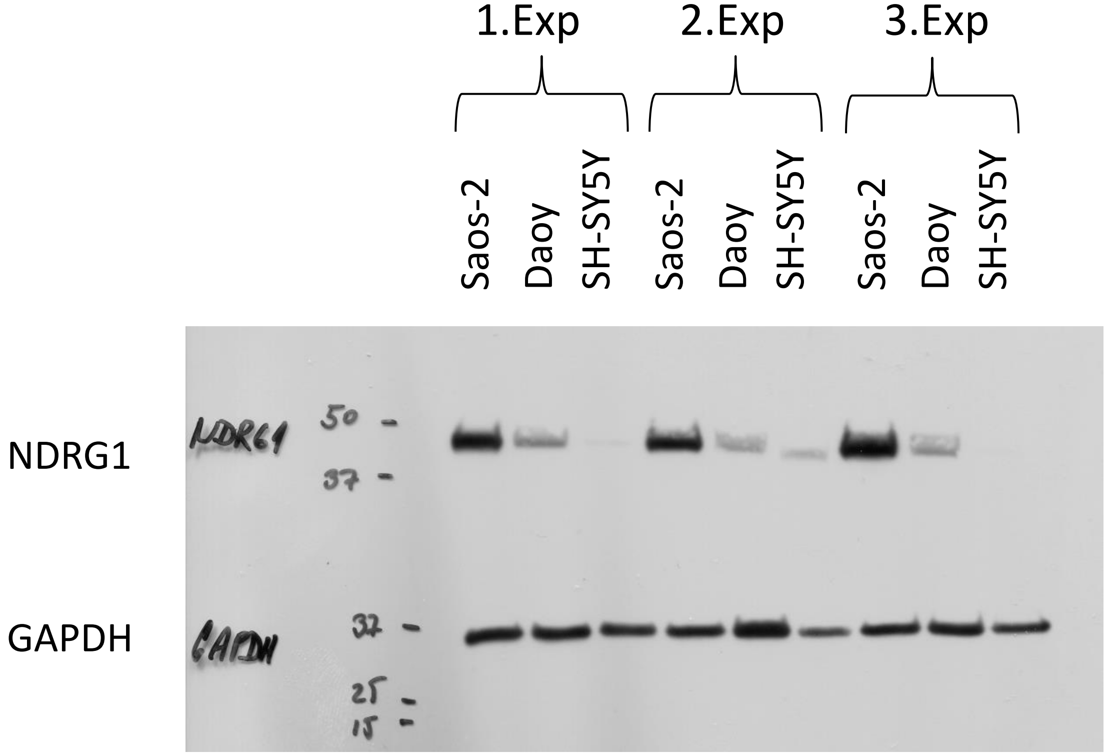

SUPPLEMENT 2A: NDRG1 levels in untreated cell lines.

Western blot analysis of the endogenous NDRG1 levels in untreated cell lines. GAPDH served as a loading control. The experiment was performed in biological triplicate.

SUPPLEMENT 2B

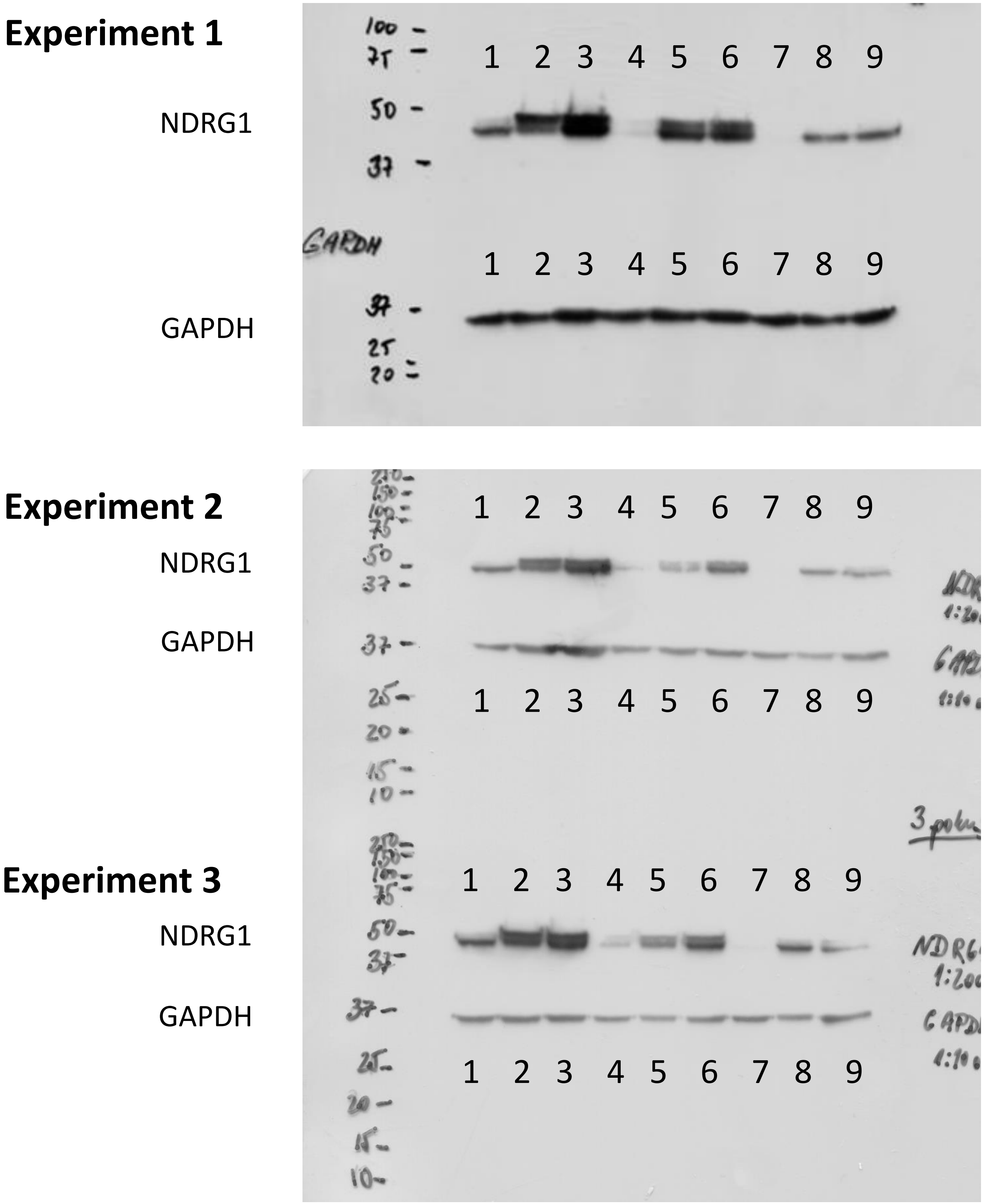

1 – Saos-2:control 2 – Saos-2: DpC 20 µM 3 – Saos-2: Dp44mT 20 µM
4 – Daoy: control 5 – Daoy: DpC 10 µM 6 – Daoy: Dp44mT 20 µM
7 – SH-SY5Y: control 8 – SH-SY5Y: DpC 20 µM 9 – SH-SY5Y: Dp44mT 20 µM

SUPPLEMENT 2B: NDRG1 levels after the treatment with thiosemicarbazones.
Western blot analysis of the NDRG1 levels in Saos-2 cells, Daoy and SH-SY5Y cells after 24 h of incubation with DpC or Dp44mT. GAPDH served as a loading control. The experiments were repeated three times.

SUPPLEMENT 3

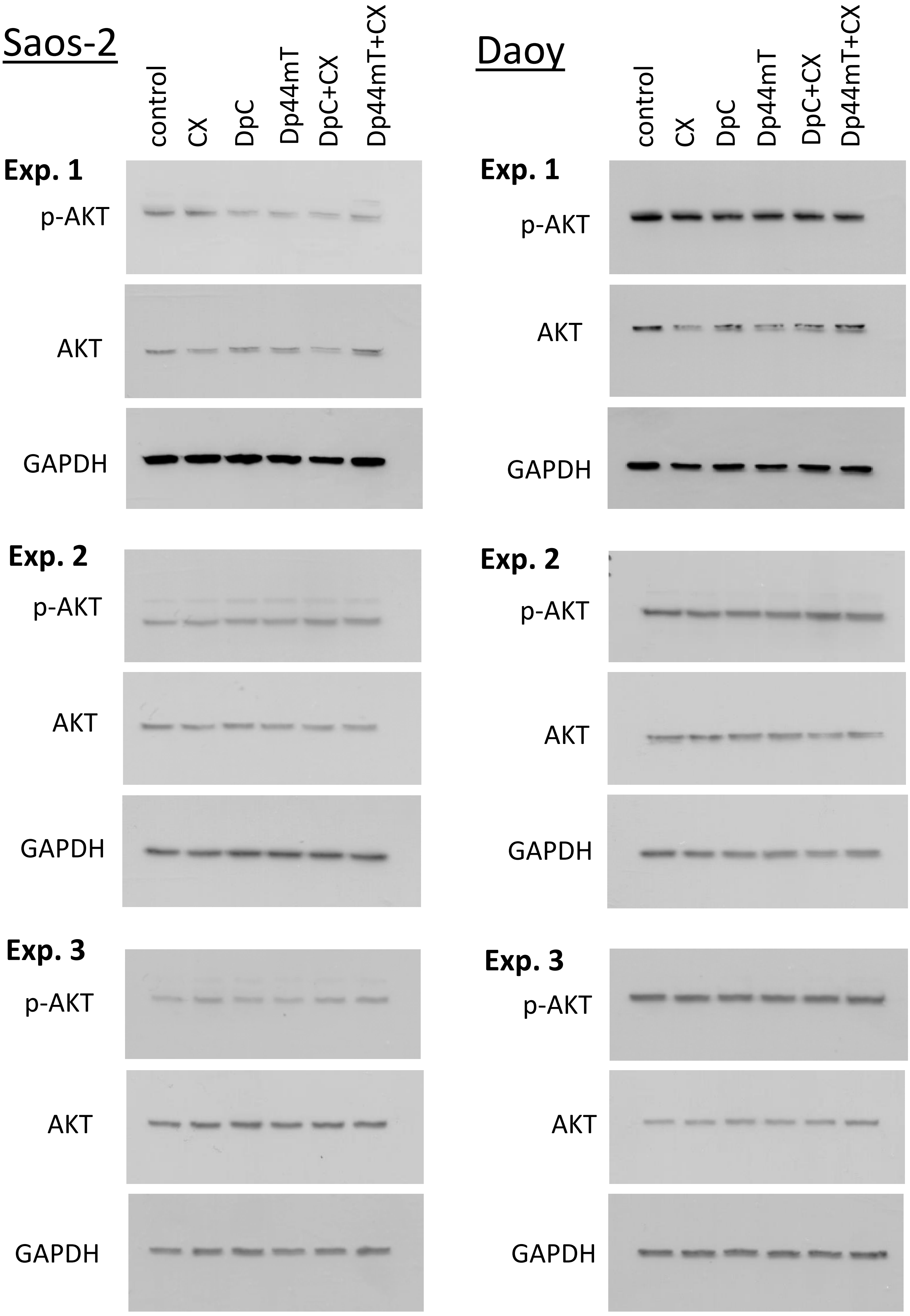

SUPPLEMENT 3

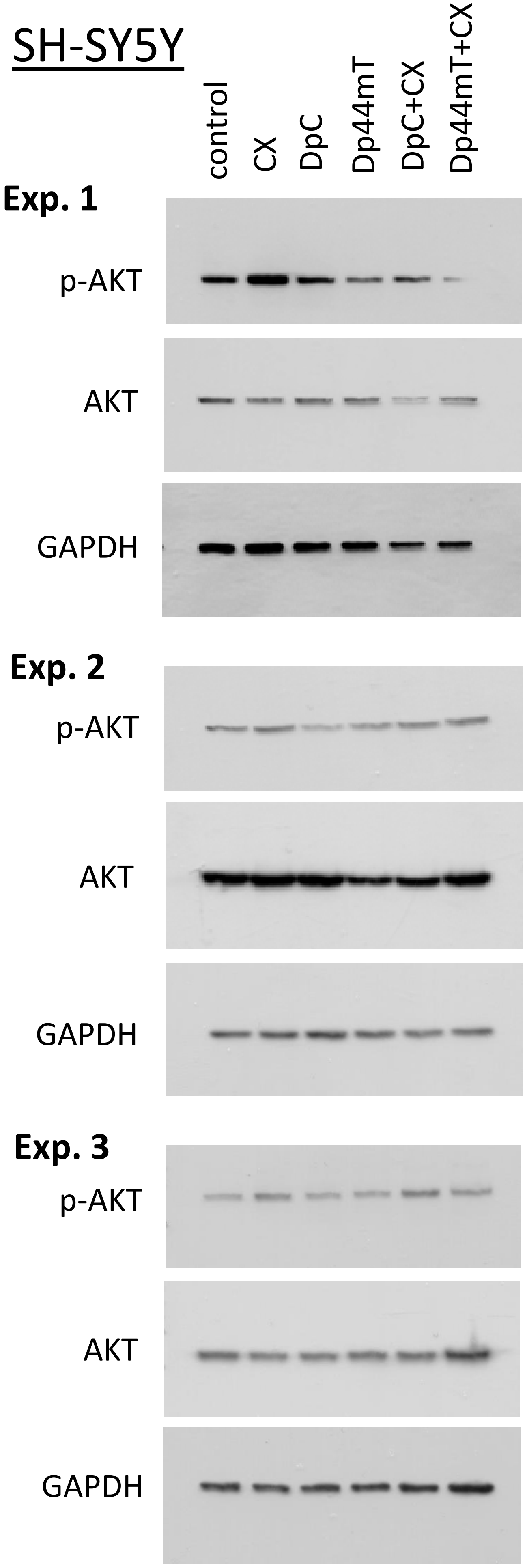

SUPPLEMENT 3: p-AKT and AKT levels after 2 h of the treatment with the IC50 doses of CX, DpC, Dp44mT and their combinations.

Western blot analysis of the phospho-AKT (p-AKT) and AKT levels in Saos-2, Daoy and SH-SY5Y cells after 2 h of incubation with the IC50 doses of CX, DpC, Dp44mT, DpC+CX or Dp44mT+CX. GAPDH served as a loading control. The experiments were performed in biological triplicates.

SUPPLEMENT 4A

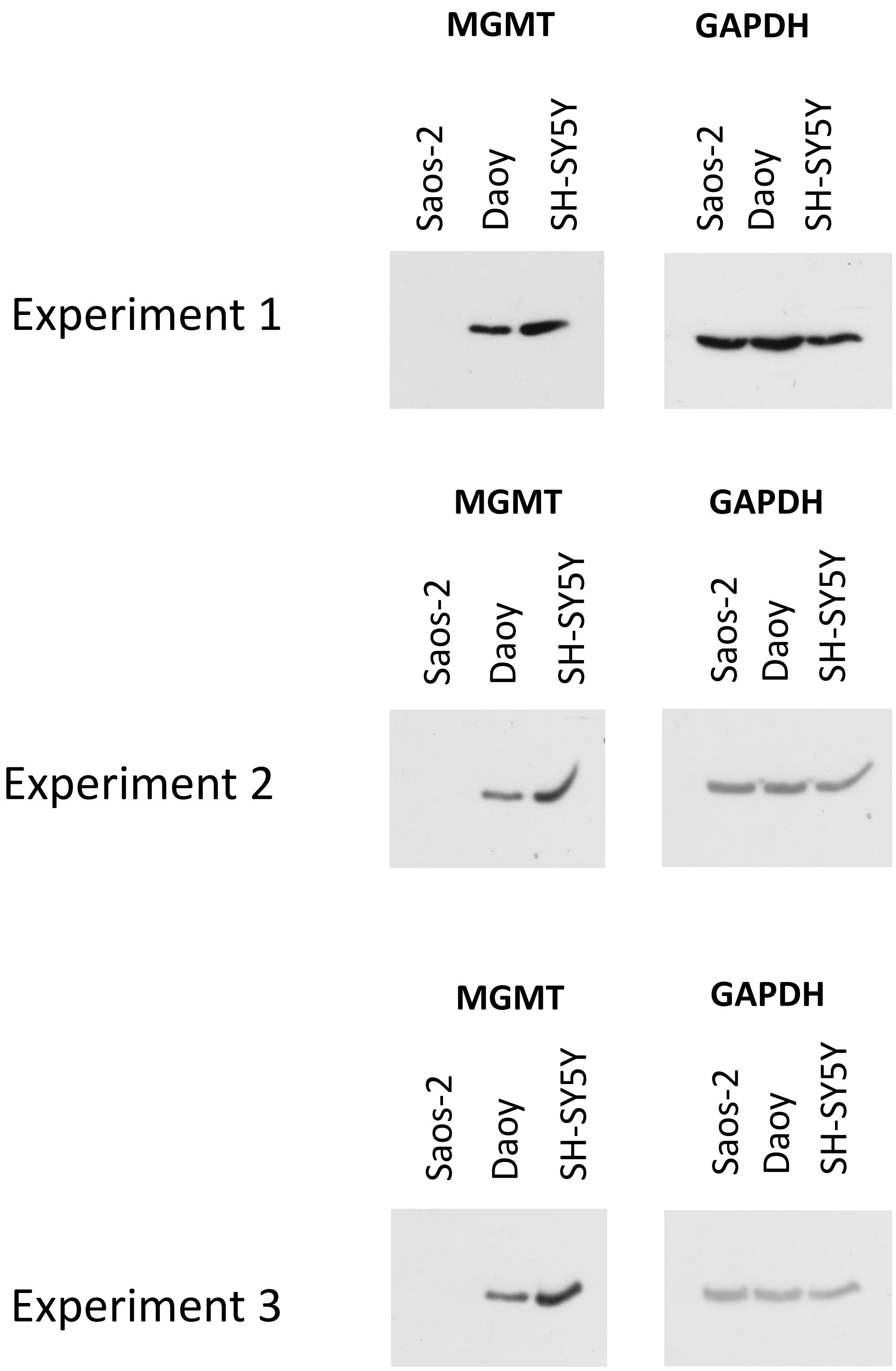

SUPPLEMENT 4A: MGMT levels in untreated cell lines.

Western blot analysis of the endogenous MGMT levels in untreated cell lines. GAPDH served as a loading control. The experiments were repeated three times.

.

SUPPLEMENT 4B

Saos-2

1 – control
2 – DpC 5 µM
3 – DpC 20 µM
4 – Dp44mT 5 µM
5 – Dp44mT 20 µM

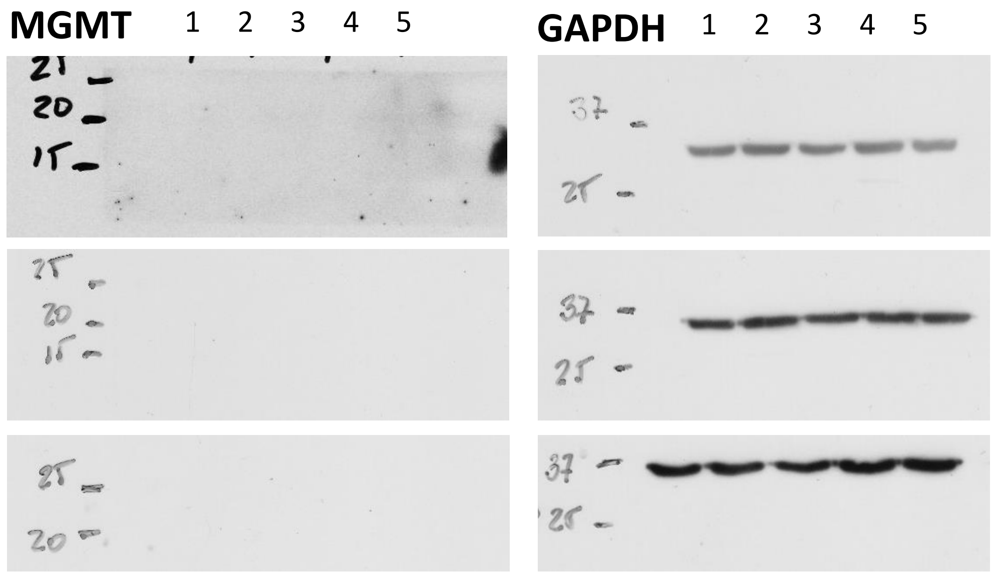

Daoy

1 – control
2 – DpC 5 µM
3 – DpC 10 µM
4 – Dp44mT 5 µM
5 – Dp44mT 20 µM

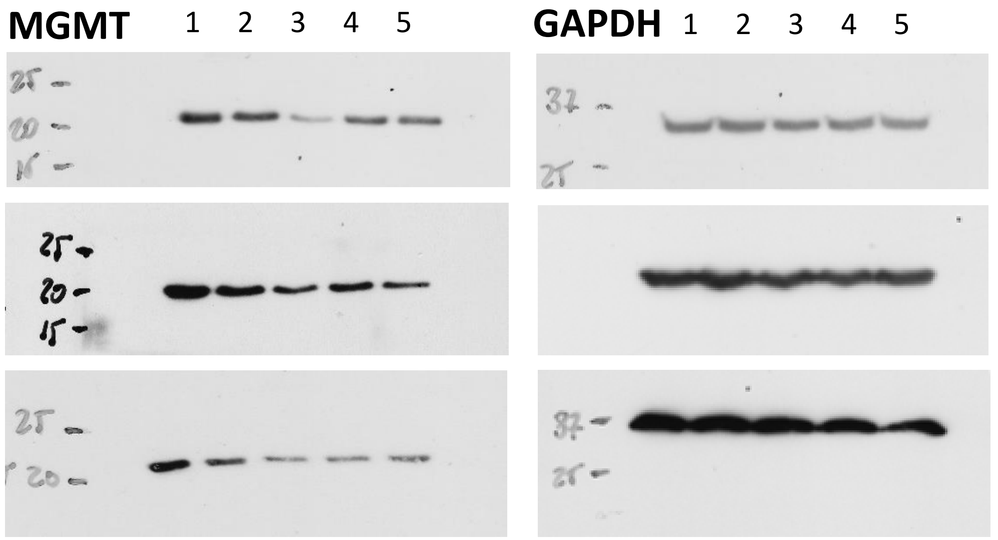

SH-SY5Y

1 – control
2 – DpC 5 µM
3 – DpC 20 µM
4 – Dp44mT 5 µM
5 – Dp44mT 20 µM

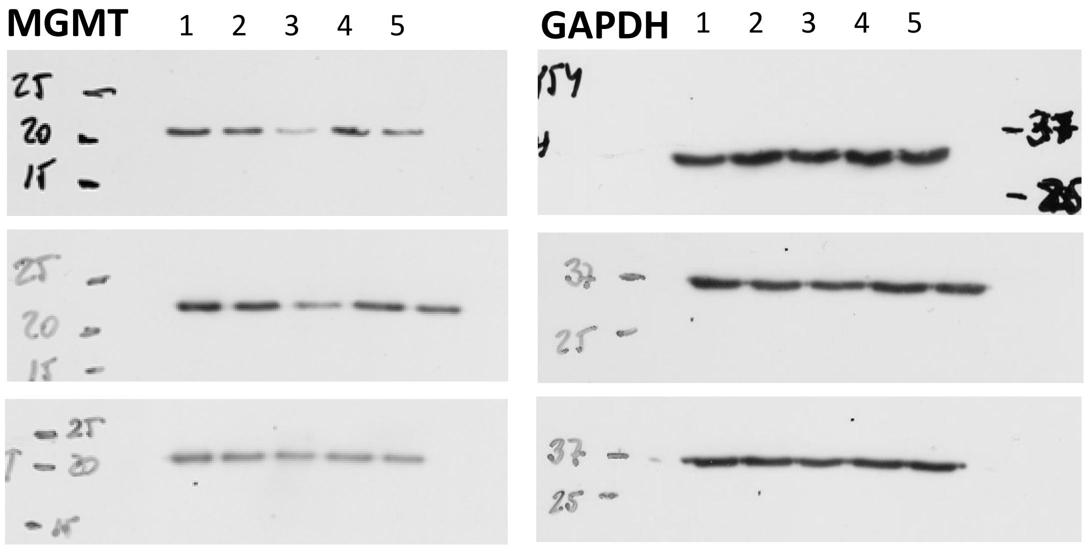

SUPPLEMENT 4B: MGMT levels after the treatment with thiosemicarbazones.

Western blot analysis of the MGMT levels in Saos-2, Daoy and SH-SY5Y cells after 24 h of incubation with DpC or Dp44mT. GAPDH served as a loading control. The experiments were repeated three times.

SUPPLEMENT 5A

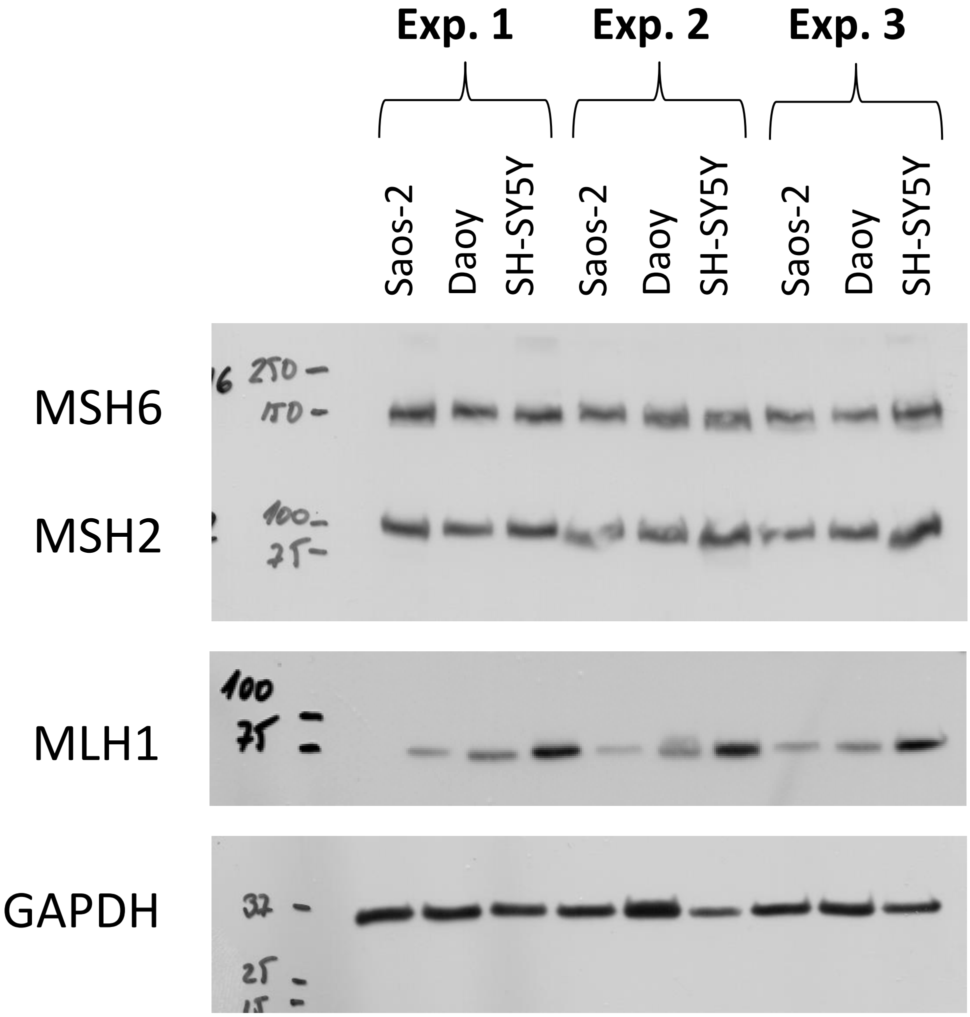

SUPPLEMENT 5A: Levels of the mismatch repair proteins in untreated cell lines.
Western blot analysis of the endogenous MLH1, MSH2 and MSH6 levels in untreated cell lines. GAPDH served as a loading control. The experiment was performed in biological triplicates.

SUPPLEMENT 5B

Experiment 1

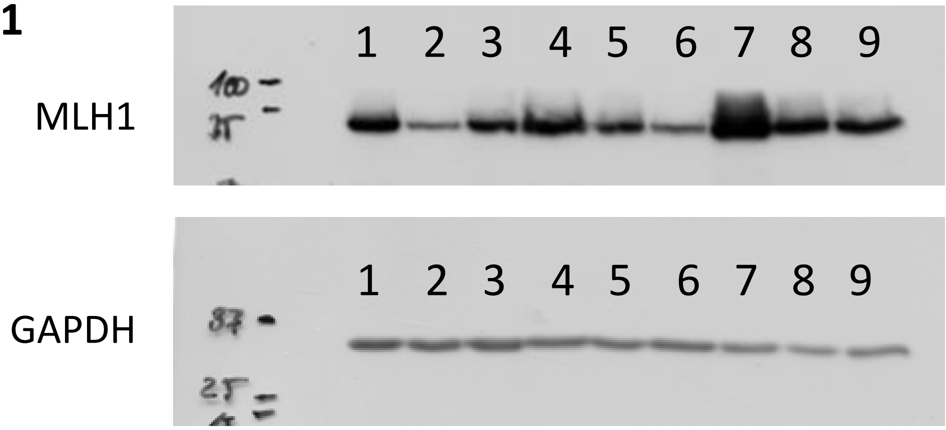

Experiment 2

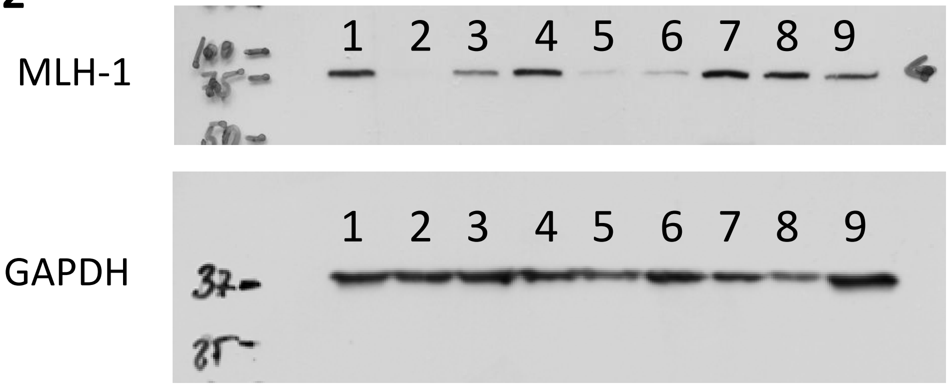

Experiment 3

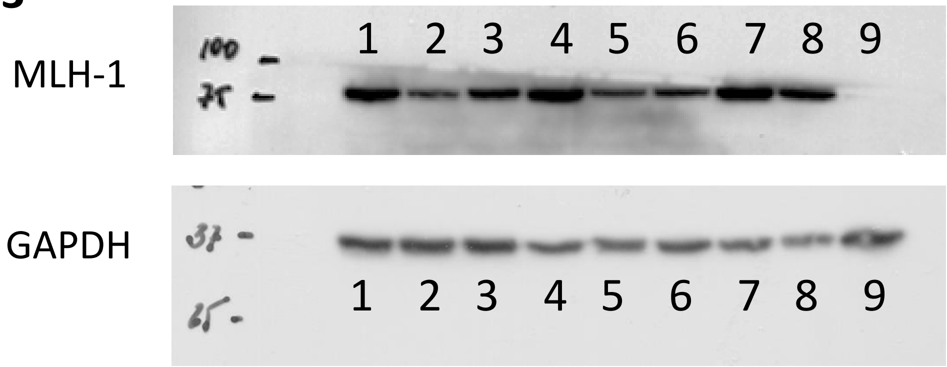

1 – Saos-2: control
2 – Saos-2: DpC 20 µM
3 – Saos-2: Dp44mT 20 µM
4 – Daoy: control
5 – Daoy: DpC 10 µM
6 – Daoy: Dp44mT 20 µM
7 – SH-SY5Y: control
8 – SH-SY5Y: DpC 20 µM
9 – SH-SY5Y: Dp44mT 20 µM

SUPPLEMENT 5B: MLH1 levels after the treatment with thiosemicarbazones

Western blot analysis of the MLH1 levels in Saos-2, Daoy and SH-SY5Y cells after 24 h of incubation with DpC or Dp44mT. GAPDH served as a loading control. The experiments were repeated three times.

SUPPLEMENT 5C

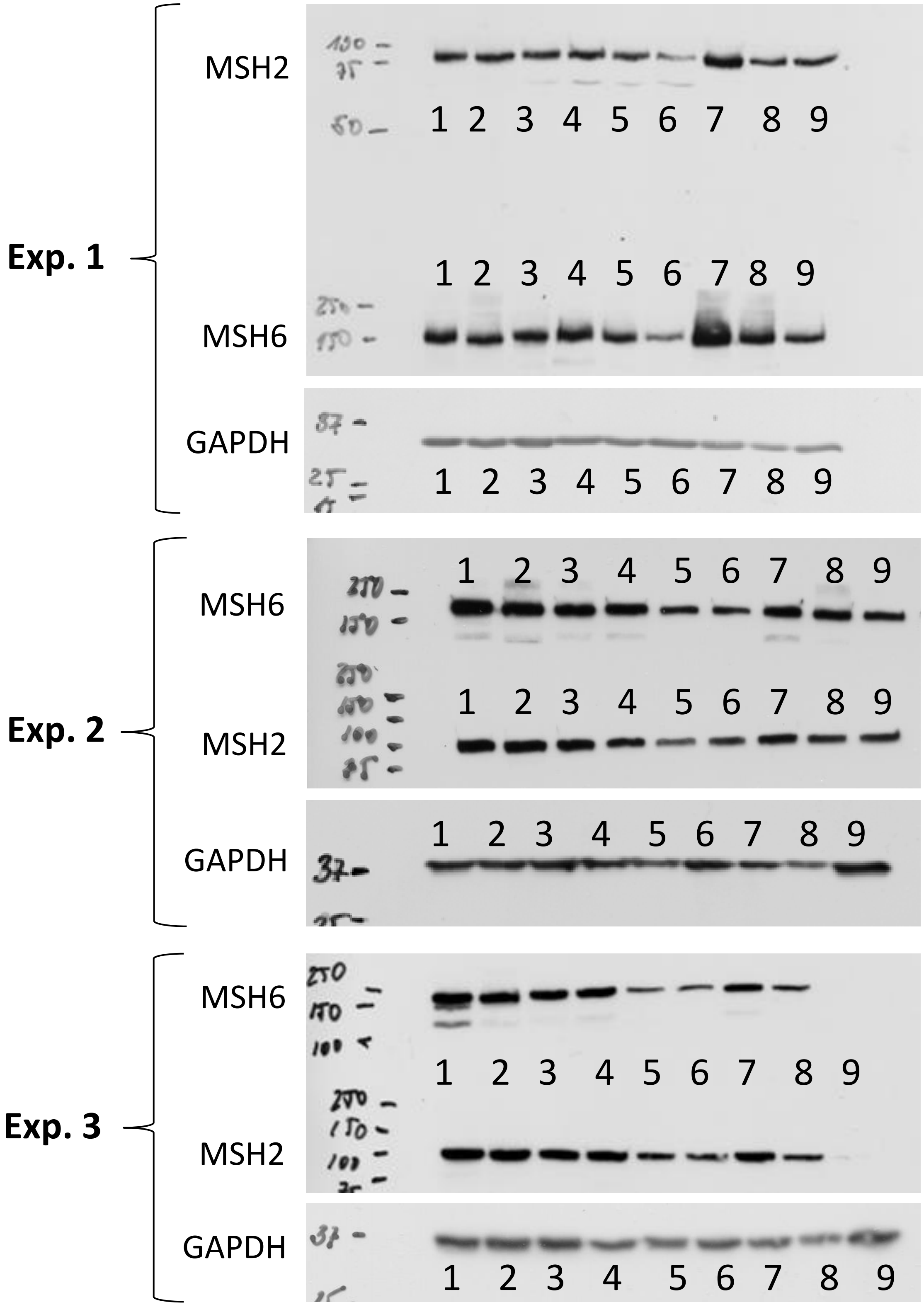

1 – Saos-2:control
2 – Saos-2: DpC 20 μM
3 – Saos-2: Dp44mT 20 μM
4 – Daoy: control
5 – Daoy: DpC 10 μM
6 – Daoy: Dp44mT 20 μM
7 – SH-SY5Y: control
8 – SH-SY5Y: DpC 20 μM
9 – SH-SY5Y: Dp44mT 20 μM

SUPPLEMENT 5C: MSH2 and MSH6 levels after the treatment with thiosemicarbazones.

Western blot analysis of MSH2 and MSH6 levels in Saos-2, Daoy and SH-SY5Y cell lines after 24 h of incubation with DpC or Dp44mT. GAPDH served as a loading control. The experiments were repeated three times.

SUPPLEMENT 6A

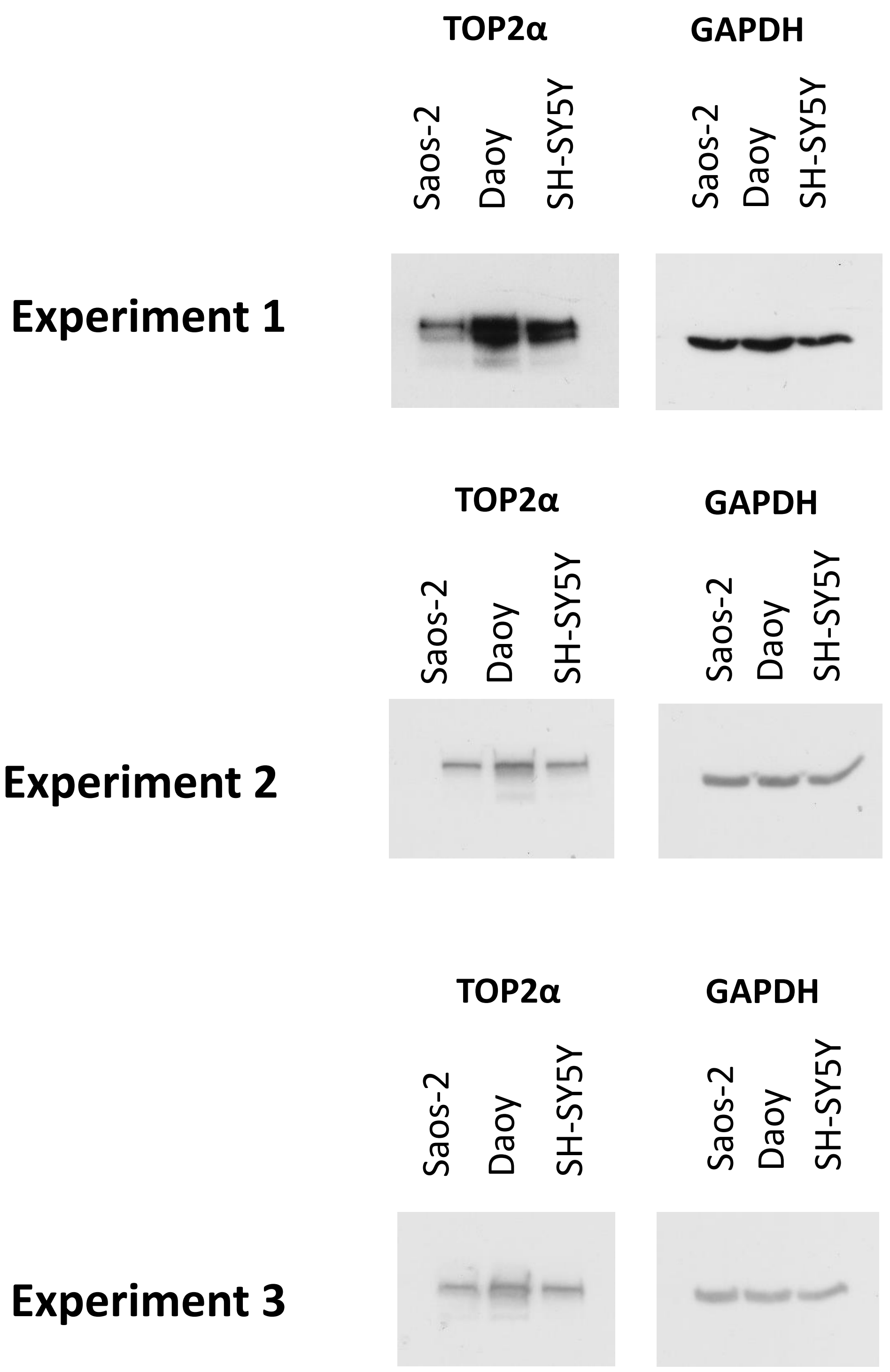

SUPPLEMENT 6A : TOP2α levels in untreated cell lines.

Western blot analysis of the endogenous TOP2α levels in untreated cell lines. GAPDH served as a loading control. The experiments were repeated three times.

SUPPLEMENT 6B

Saos-2

1 – control
2 – DpC 5 µM
3 – DpC 20 µM
4 – Dp44mT 5 µM
5 – Dp44mT 20 µM

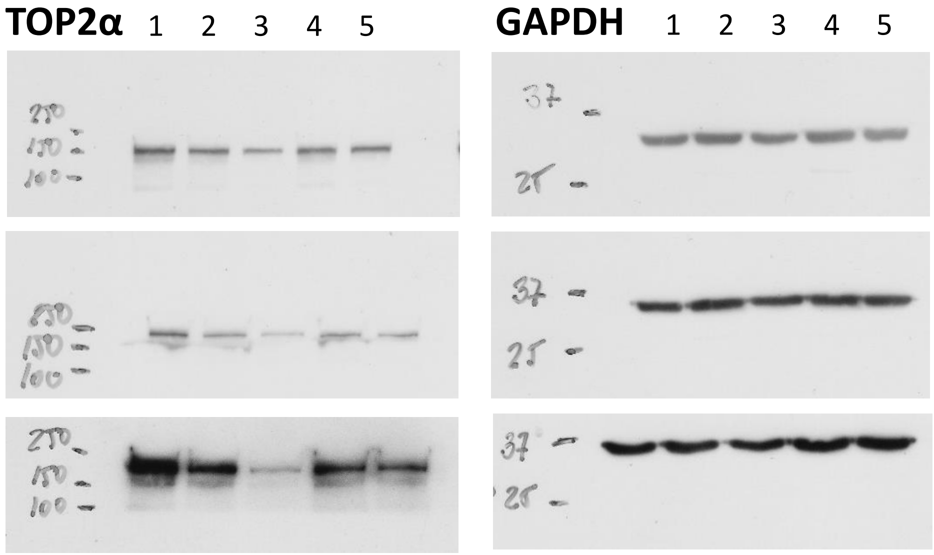

Daoy

1 – control
2 – DpC 5 µM
3 – DpC 10 µM
4 – Dp44mT 5 µM
5 – Dp44mT 20 µM

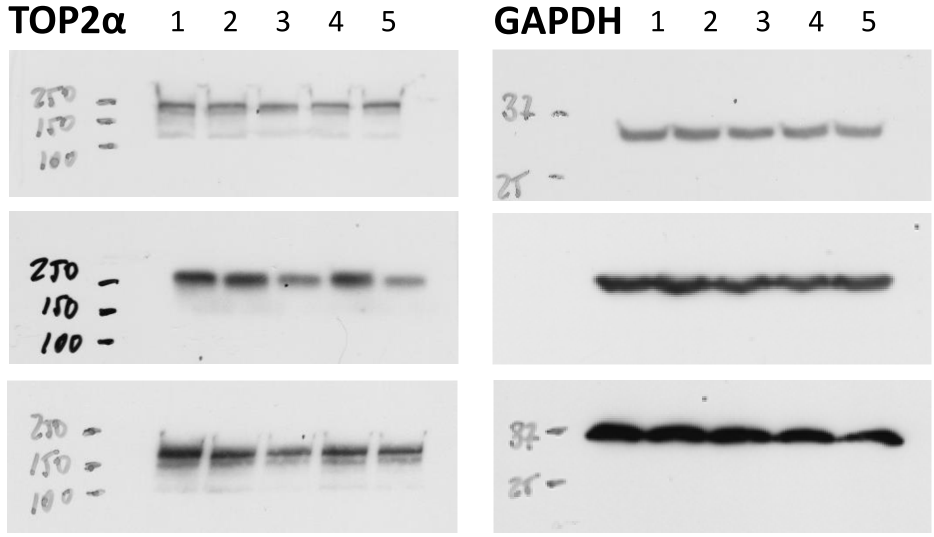

SH-SY5Y

1 – control
2 – DpC 5 µM
3 – DpC 20 µM
4 – Dp44mT 5 µM
5 – Dp44mT 20 µM

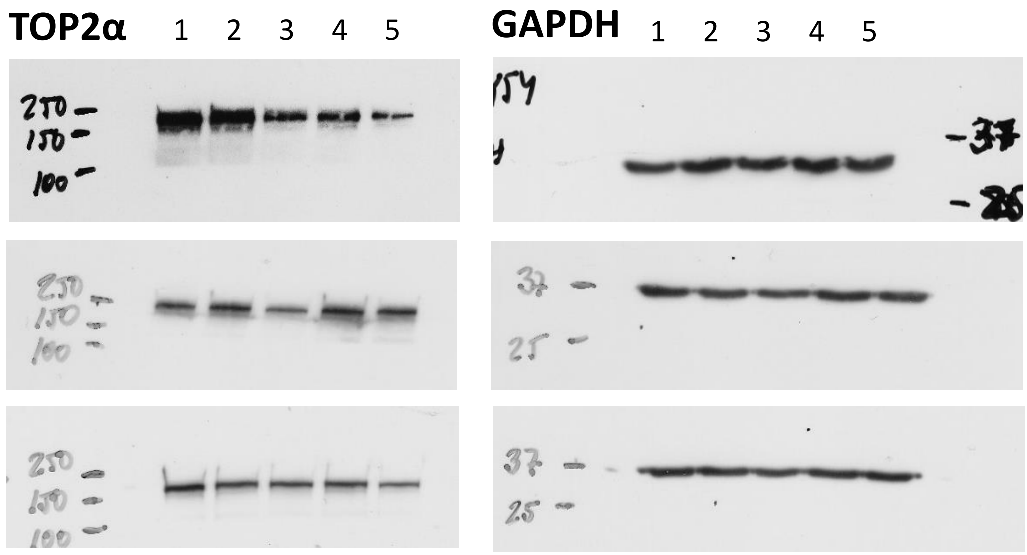

SUPPLEMENT 6B: TOP2α levels after the treatment with thiosemicarbazones.

Western blot analysis of the TOP2α levels in Saos-2, Daoy and SH-SY5Y cells after 24 h of incubation with DpC or Dp44mT. GAPDH served as a loading control. The experiments were repeated three times.
